# Phylogenetic analysis of *Spirocerca lupi* and *Spirocerca vulpis* reveal high genetic diversity and intra-individual variation

**DOI:** 10.1186/s13071-018-3202-0

**Published:** 2018-12-14

**Authors:** Alicia Rojas, Eran Dvir, Róbert Farkas, Kalyan Sarma, Sonjoy Borthakur, Abdul Jabbar, Alex Markovics, Domenico Otranto, Gad Baneth

**Affiliations:** 1 Koret School of Veterinary Medicine, The Hebrew University of Jerusalem, Rehovot, Israel; 2 Department of Animal Sciences, Tel-Hai Academic College, Upper Galilee, Israel; 3 Department of Parasitology and Zoology, University of Veterinary Medicine, Budapest, Hungary; 4 Department of Veterinary Medicine, College of Veterinary Sciences and Animal Husbandry, Central Agricultural University, Mizoram, India; 5 Department of Veterinary Parasitology, College of Veterinary Sciences and Animal Husbandry, Central Agricultural University, Mizoram, India; 6 Department of Veterinary Biosciences, Melbourne Veterinary School, The University of Melbourne, Victoria, Australia; 7 Kimron Veterinary Institute, Bet Dagan, Israel; 8 Department of Veterinary Medicine, University of Bari, Valenzano, Italy

**Keywords:** *Spirocerca lupi*, *Spirocerca vulpis*, ITS1, *cox*1, *18S*, Genetic variation

## Abstract

**Background:**

*Spirocerca lupi* is a parasitic nematode of canids that can lead to a severe and potentially fatal disease. Recently, a new species, *Spirocerca vulpis*, was described from red foxes in Europe, suggesting a high genetic diversity of the *Spirocerca* spp. infecting canids. The genetic variation and phylogenetic relationships of *S. lupi* collected from naturally-infected domestic dogs from Australia, Hungary, Israel, Italy, India and South Africa, and *S. vulpis* from red foxes from Bosnia and Herzegovina, Italy and Spain, was studied using mitochondrial and rDNA markers.

**Results:**

A high intra-individual variation was found in the first internal transcribed spacer (ITS1) locus in all *Spirocerca* spp., ranging between 0.37–2.84%, with up to six haplotypes per specimen. In addition, a combination of phylogenetic and haplotype analyses revealed a large variability between *S. lupi* specimens collected from different geographical locations using the ITS1 (0.37–9.33%) and the cytochrome *c* oxidase subunit 1 (*cox*1) gene (1.42–6.74%). This genetic diversity led to the identification of two *S. lupi* genotypes circulating among dogs (PTP support > 0.829), including genotype 1 found in *S. lupi* from Australia, India, Israel and South Africa, and genotype 2 represented by specimens from Hungary and Italy. These genotypes presented pairwise nucleotide distances of 0.14%, 8.06% and 6.48 ± 0.28% in the small rDNA subunit (*18S*), ITS1 and *cox*1 loci, respectively. Additionally, Nei’s genetic distance in the ITS1 showed a further subdivision of genotype 1 worms into 1A (Israel and South Africa) and 1B (Australia and India). A morphological analysis of the anterior and posterior extremities of genotype 1 and genotype 2 worms using scanning electron microscopy did not show any differences between the specimens, contrary to the morphological differences between *S. lupi* and *S. vulpis*.

**Conclusions:**

These findings demonstrate the high genetic variability among *Spirocerca* spp. from different geographical locations, thereby expanding our understanding of the epidemiology, evolution and phylogenetic variability within the genus.

**Electronic supplementary material:**

The online version of this article (10.1186/s13071-018-3202-0) contains supplementary material, which is available to authorized users.

## Background

*Spirocerca lupi* is a parasitic nematode that affects canids in tropical and subtropical regions around the world [1]. Its life-cycle includes coprophagous beetles and canids, as intermediate and definitive hosts, respectively. Among canids, dogs (*Canis lupus familiaris*) are considered the main definitive hosts, while other wild canid species have been reported as potential alternative hosts for this parasite [2–4]. In dogs, *S. lupi* infection leads to spirocercosis, a life-threatening disease with clinical signs that range from regurgitation and vomiting of food, fatal hemothorax due to rupture of aortic aneurisms, neurological signs of spinal pain and paralysis due to spinal migration of the worm, and malignant transformation of oesophageal nodules with metastasis to other organs [1]. Recently, a new species of the genus *Spirocerca* was described from red foxes (i.e. *Spirocerca vulpis*), which is morphologically as well as genetically different from *S. lupi* and produces stomach nodules in these hosts [5]. The presence of this additional species in canids previously thought to be *S. lupi* highlights the need to further study the genetic composition and variability of this species.

Genetic diversity has been widely studied in other helminth species, such as *Fasciola hepatica*, *Sabatieria* spp. and *Gongylonema pulchrum* [6–8], leading to important implications about the evolutionary history and epidemiology dynamics of helminth infections. In the case of *S. lupi*, contrasting results have been obtained regarding the genetic composition in specimens from different parts of the world. For example, Traversa et al. [9] found low genetic variability in specimens of *S. lupi* obtained from Africa, Asia and Europe. In contrast, a study conducted in a limited area in South Africa found a high intra-host genetic variability and low variation within the analyzed area [10]. Similarly, a high genetic diversity was recently reported in *S. lupi* obtained from dogs and black backed jackals in South Africa [2] and from the Andean fox in Peru [11]. Furthermore, the analysis of three *S. lupi* populations from South Africa found a low population structure between groups and lack of panmixia [12]. The latter studies led to the suggestion of *S. lupi* as a species complex or the presence of cryptic and/or different *Spirocerca* spp. circulating among canid populations around the world [13]. In this regard, geographically isolated canid populations may lead to the genetic differentiation and divergence of parasite species through time [14]. Therefore, the genetic comparison of parasites obtained from different geographical locations or host species can assist in the delimitation of novel or cryptic species [15]. Thus, a deeper exploration of the genetic diversification of *S. lupi* and *S. vulpis* collected from different parts of the world is required to decipher the phylogenetic relationships between these specimens and determine the degree of genetic variability.

Mitochondrial and nuclear ribosomal DNA (rDNA) markers have extensively been used for phylogenetic and diversity studies in several parasite species [15, 16]. The rRNA genes are usually encoded in the genome in multiple tandem copies, which include conserved coding regions (i.e. *18S*, *5.8S* and *28S* genes) separated by highly variable non-coding spacers known as the first and second internal transcribed spacers (ITS1 and ITS2, respectively) or intergenic spacers [17]. In helminths, the characterization of the ITS1 and ITS2 loci has shown a high variability between species within the same genera [18], between individuals of the same species [19] or even within a single adult worm [20, 21]. This characteristic makes the ITS sequences suitable targets for phylogenetic comparisons among nematodes of the same species from different geographical and host origins [22]. However, the ITS1 of *S. lupi* has not been characterized to date and no analyses comparing specimens from different countries have been assessed.

The purpose of the present study was to analyze the phylogeny, genetic variability and diversity of *S. lupi* from dogs collected from six countries in four different continents, and *S. vulpis* obtained from red foxes collected from three European countries, using three different genetic markers (i.e. ITS1, *18S* and *cox*1). This study reveals the existence of two major *S. lupi* genotypes distributed according to geographical locations and shows the high genetic diversity present in the genus*.* Overall, these results highlight the utility of these markers for further genetic population and evolutionary studies.

## Methods

### Specimen collection and DNA extraction

A total of 31 *S. lupi* and *S. vulpis* specimens were obtained from dogs and red foxes from eight countries (Table 1). All the specimens from dogs were obtained from oesophageal nodules after *post-mortem* dissection of the animals. The worms from foxes were collected from nodules localized in the stomach wall from authorized captures, including wildlife protection centers or from road-killed animals after car accidents. Details on the year of collection, the number of worms processed from each collection site and the code assigned for each worm specimen are included in Table 1.

**Table 1:** Summary of data on *Spirocerca* spp. adult worms included in the study

Species	Country of origin	Year of collection	Host	No. of specimens	Code
*S. lupi*	Israel	1993	Dog	3	A, G, H^a^
		2004	Dog	1	B
		2013	Dog	1	C
		2015	Dog	3	D, E, F^a^
	South Africa	2009	Dog	3	I, J, K
		2010	Dog	1	L
	India	2016	Dog	5	M, N, O, P, Q
	Italy	2013	Dog	1	R
	Australia	1989	Dog	2	V, W^a^
	Hungary	2016	Dog	4	AA, BA, CA, DA^a^
*S. vulpis*	Spain	2015	Red fox	3	S, T, U
	Italy	2015	Red fox	1	X
	Bosnia and Herzegovina	2016	Red fox	3	EA, FA, GA

^a^These worms are siblings, i.e. were recovered from the same dog

DNA was extracted from approximately 1 cm of the worms’ body using the DNeasy Blood & Tissue kit (Qiagen, Hilden, Germany) following the manufacturer’s instructions.

### Amplification of the *18S*, ITS1 and *cox*1 loci

The target loci, PCR primers and their concentrations, and the conditions for PCR amplification are specified in Table 2 and in Additional file 1: Figure S1. All reactions for amplifying the *18S*, ITS1 and *cox*1 loci were run in a final volume of 25 μl containing primers at different concentrations, 150 ng of DNA template, and 20 μl of ultra-pure water (UPW) in ready-to-use PCR tubes containing a dehydrated master mix (Syntezza Bioscience Ltd., Jerusalem, Israel). In addition, all PCRs in this study included positive (*S. lupi-*DNA), negative (*Leishmania infantum-*DNA) and non-template (UPW) controls. All amplicons obtained were examined on 2% agarose gels stained with ethidium bromide. Accordingly, a 1611 bp fragment of the *18S* gene was obtained by joining the outcomes of two separate conventional PCRs using two different sets of primers (Table 2, Additional file 1: Figure S1) [23].

**Table 2:** Summary of the gene targets, primers, PCR conditions employed for the amplification and genetic analysis of *Spirocerca* spp.; and the outgroups used for the phylogenetic analyses

Gene target	Amplicon length (bp)	Primers (final concentration used in the PCR) [reference]	PCR conditions	Outgroups employed in phylogenetic analysis
*18S*	~750	Nem18S-F (5'-CGC GAA TRG CTC ATT ACA ACA GC-3') and Nem18S-R (5'-GGG CGG TAT CTG ATC GCC-3') (400 nM) [23]	95 °C for 5 min; 35 cycles of 95 °C for 1 min, 56 °C for 1 min and 72 °C for 1 min; 72 °C for 5 min	*Spirocerca lupi* (AY751497.1 and Q674750.1), *Spirocerca* sp. (AY751498.1), *Cylicospirura petrowi* (KM434335.1), *Gongylonema pulchrum* (AB495401.2), *Gongylonema nepalensis* (AB646109.1), *Oxyspirura petrowi* (LC316613.1), *Dirofilaria immitis* (AF182647.1), *Dirofilaria repens* (AB973229.1), *Loa loa* (XR002251421.1), *Wuchereria bancrofti* (AY843436.1), *Litosomoides sigmodontis* (AF227233.1), *Thelazia lacrymalis* (DQ503458.1), *Thelazia callipaeda* (AB538282.1)
	~870	Nem18S-F2 (5'-CGA AAG TCA GAG GTT CGA AGG-3') and Nem18S-R2 (5'-AAC CTT GTT ACG ACT TTT GCC C-3') (400 nM) [5]	95 °C for 5 min; 35 cycles of 95 °C for 1 min, 56 °C for 1 min and 72 °C for 1 min; 72°C for 5 min	
ITS1	610	rDNA2 (5'-TTG ATT ACG TCC CTG CCC TTT-3') [24] and rDNA1.58S (5'-GCC ACC TAG TGA GCC GAG CA-3') (375 nM) [25]	94 °C for 5 min; 35 cycles of 94 °C for 1 min, 60 °C for 1 min and 72 °C for 2 min; 72 °C for 5 min	*C. petrowi* (KM434335.1)
	~100	ITS inner-F (5'-GCT ATC TTG TAA AAA CGG TG-3')^a^ and M13-R (5'-CAG GAA ACA GCT ATG AC-3') (200 nM)	94 °C for 5 min; 35 cycles of 94 °C for 1 min, 60 °C for 1 min and 72 °C for 2 min; 72 °C for 5 min	
	450	C18 (5'-GTT TCC GTA GGT GAA CCT GC-3') and 5818 (5'-ACG ARC CGA GTG ATC CAC-3') (400 nM) [26]	94 °C for 4 min; 40 cycles of 94 °C for 45 s, 59 °C for 30 s and 72 °C for 2 min; 72 °C for 5 min	
*cox*1	650 (Fragment A)	NTF (5'-TGA TTG GTG GTT TTG GTA A-3') and NTR (5'-ATA AGT ACG AGT ATC AAT ATC-3') (200 nM) [28]	95 °C for 2 min; 49 cycles of 95 °C for 1 min, 54 °C for 1 min, 72 °C for 1 min; 72 °C for 7 min	*S. lupi* from China (KC305876.1) [27], *Spirocerca* sp. from Denmark (KJ605484) [13], *C. petrowi* (KF719952.1), *Cylicospirura felineus* (GQ342967.1) [61], *Cylicospirura subaequalis* (GQ342968.1) [60]
	~300	NTInt (5'-GGC TAG ACA ACT CTA AAC G-3')^a^ and NTF (5'-TGA TTG GTG GTT TTG GTA A-3') (200 nM) [28]	95 °C for 2 min; 49 cycles of 95 °C for 1 min, 54 °C for 1 min, 72 °C for 1 min; 72 °C for 7 min	
	394 (Fragment B)	JB3 (5'-TTT TTT GGG CAT CCT GAG GTT TAT-3') and JB4.5 (5'-TAA AGA AAG AAC ATA ATG AAA ATG-3') (400 nM) [30]	95 °C for 5 min; 30 cycles of 95 °C for 1 min, 54 °C for 1 min and 72 °C for 1 min; 72 °C for 5 min	*S. lupi* from China (KC305876.1) [27], *S. lupi* obtained from domestic dogs and black-backed jackals from South Africa (KY495493.1-KY495505.1) [2] and from the Andean fox from Peru (KY634868.1-KY634870.1) [11]

^a^This primer was designed in the present study using Primer-BLAST [29]

The complete ITS1 locus of the *Spirocerca-*adults was amplified by conventional PCR using primers rDNA2 [24] and rDNA1.58S [25] (Table 2, Additional file 1: Figure S1). In case of negative results in this reaction, an additional PCR was performed using the C18 and 5818 primers [26] (Table 2). Preliminary sequencing of the ITS1 amplicons, suggested the presence of multiple different ITS1 copies within each specimen (i.e. unresolved chromatograms). Thus, all ITS1 PCR amplicons were cloned into plasmids in competent bacterial cells for further individual amplification and sequencing. Accordingly, the amplicons were excised and purified from agarose gels using the NucleoSpin® Gel and PCR Clean-up kit (Macherey-Nagel GmbH & Co., Düren, Germany). Subsequently they were cloned into pCR 2-TOPO vectors using the Invitrogen TOPO TA cloning kit (Life Technologies, Thermo Fisher Scientific Inc, Waltham, USA) with the white-blue colony screening protocol, according to the manufacturer’s instructions. To allow determination of different ITS1 copies per individual, plasmids were extracted from 12 different colonies per nematode using the FB Plasmid Miniprep kit (FairBiotech Corp., Taoyuan, China). Subsequently, the cloned fragments were amplified by conventional PCR using the universal primers M13-F and M13-R provided with the kit. In cases where unresolved sequences were obtained, the ITS1 clones were further tested in a conventional PCR with the designed primer ITS inner-F and M13-R (Table 2). Finally, the amplicons were sequenced as described below.

The *cox*1 gene was amplified using different primer sets to allow the comparison of the study specimens with sequences from different organisms available on GenBank, as follows. First, a 650 bp fragment (positions 317 to 967 bp of the *cox*1 gene; [27], hereinafter referred to as *cox*1 fragment A) was amplified using the NTF and NTR primers [28] (Table 2, Additional file 1: Figure S1), which allowed comparison with some *Spirocerca* spp. and other *Cylicospirura* spp. sequences. Larger than expected amplicons (~1000 bp) were obtained from several specimens, which amplified an additional 300 bp-upstream region from the *cox*1 fragment A (demonstrated by sequencing analysis; Additional file 1: Figure S1, Additional file 2: Figure S2). To confirm the latter, a reverse primer, identified as NTInt was designed using Primer-BLAST [29] and run together with the NTF primer in an additional PCR (Table 2). Secondly, a 394 bp fragment from positions 807 to 1201 bp of the *cox*1 gene (hereinafter referred to as *cox*1 fragment B) was amplified using primers JB3 and JB4.5 [30] (Table 2, Additional file 1: Figure S1) to compare the study’s sequences with other *S. lupi* sequences available on GenBank.

### DNA sequencing of amplicons

All DNA amplicons were purified using the Exo-SAP mix (New England Bio-Labs Inc., Ipswich, USA) and sequenced using primers in both directions in the Big-Dye Terminator cycle sequencing chemistry from Applied Biosystems ABI3700 DNA Analyzer and the ABI’s Data Collection and Sequence Analysis software (Applied Biosystems, Thermo Fisher Scientific Inc., Waltham, USA).

### Sequence analyses

All sequences were manually inspected, cleaned of nucleotide ambiguities and trimmed from the primer sequences using the MEGA 7.0 software [31]. Each indel and/or single nucleotide substitution present in the sequences were double-checked by manual verification in the sequence chromatogram and re-sequenced in case of ambiguity. The *18S*, ITS1 and *cox*1 sequences obtained in this study were aligned together with sequences for other *Spirocerca* spp. and nematode outgroups (specified in Table 2), in accordance with their GenBank availability, using the ClustalW algorithm with conventional gap opening and extension penalty of 15 and 6.66.

The boundaries of the ITS1 of *Spirocerca* spp. (i.e. the *18S* downstream and the *5.8S* upstream regions) were determined using the outgroup sequences (Table 2) and *Thelazia* spp. sequences [32]. The G+C content was calculated using the ENDMEMO webtool (http://www.endmemo.com/bio/gc.php) and microsatellites were searched for using the Microsatellite repeats finder webtool (http://insilico.ehu.es/mini_tools/microsatellites/) with 2 to 6 bp sequence length and a minimum of 4 repeats. An unpaired t-test (Excel 365 software, Microsoft, Redmont, USA) was employed to determine differences in the length and G+C content of ITS1 sequences obtained from different specimens of *Spirocerca* spp. The Bonferroni correction was applied in multiple comparisons and significance was determined when *P* < 0.003.

Both overlapping *cox*1 fragments obtained in our specimens were assembled to generate an 888 bp contiguous sequence, which was compared to *D. immitis* (GenBank: AJ537512.1) and used for further analyses.

### Genetic diversity, differentiation and distance analyses

The number of haplotypes (*h*), haplotype diversity (Hd), segregating sites (S), and nucleotide diversity (π) were calculated for the ITS1 and the 888 bp-assembled *cox*1 fragment using DnaSP 5.10.01 [33] with 10,000 permutations. The gaps generated in the ITS1 alignment were not included in this analysis. Additionally, the ITS1 and *cox*1 genetic distances between sampled individuals was calculated separately according to Nei [34]. A principal coordinate analysis (PCoA) was drawn using the GenAIEx 6.5 software [35]. Furthermore, the pairwise nucleotide p-distances of the ITS1, *cox*1 (A, B and combined fragments) and *18S* sequences were calculated using the MEGA 7.0 software [31]. Complete deletion was assumed in sites with gaps. In the ITS1 analysis, the generated sequences from each specimen were compared between each other with the median and third interquartile ranges (3rd IQR). All ITS1, *18S* and *cox*1 sequence novel genetic variants detected in this study were deposited in the GenBank database.

### Phylogenetic and species delimitation analyses

The phylogenetic relationships of the ITS1 and *cox*1 (with the 888 bp-assembled fragment) haplotypes were analyzed by building a haplotype network for each locus separately. A Templeton-Crandall-Sing (TCS) haplotype network was drawn with the method of statistical parsimony [36] and a 95% connection limit ignoring sites with gaps using the PopART software (available at http://popart.otago.ac.nz).

The ITS1, *18S* and 888 bp-*cox*1 alignments were analyzed using Bayesian inference (BI) and Maximum Likelihood (ML) phylogenetic algorithms. First, the best nucleotide substitution model for the BI tree was chosen according to the Akaike information criterion (AIC) as determined by JModelTest 2.1.6 [37] implemented on the XSEDE server of the Cipres Science Gateway [38]. Ultrametric trees were generated with the Bayesian Evolutionary Analysis by Sampling Trees (BEAST) package 1.8.4. The program Bayesian Evolutionary Analysis Utility (BEAUti) was used to generate the input XML file with a Markov Chain Monte Carlo (MCMC) analysis run for 10^7^ generations, a sampling frequency of every 10^3^ generated trees, 10^6^ states of 'burn-in' length and a relaxed lognormal clock. The nucleotide substitution models Hasegawa-Kishino-Yano with gamma distribution (HKY+G) (*γ* = 4), the three parameters with gamma distribution (TPM+G) (*γ* = 4) and the transitional model with gamma distribution (TIM+G) (*γ* = 4) were implemented for the ITS1, *18S* and *cox*1 databases, respectively. The convergence of the chains was verified by the effective sample sizes (ESS) with values larger than 300 in all priors using Tracer 1.6.0. The generated trees for all loci were summarized with TreeAnotator 1.8.4 and the consensus trees were visualized using FigTree 1.4.3. Secondly, the ML tree was generated using the PhyML online execution program (http://www.atgc-montpellier.fr/phyml/) [39] with bootstrap replicates set to 10^3^. In addition, the nucleotide substitution model was selected according to the AIC using the Smart Model Selection (SMS) in the same website [40]. The chosen models were HKY+G (*γ* = 4) for the ITS1 and *cox*1 databases, and the general time reversible with gamma distribution and invariant sites (GTR+G+I) (*γ* = 4) for the *18S* alignment. The outgroup sequences used for each tree are specified in Table 2.

Finally, the Poisson Tree Processes (PTP) [41] for species delimitation were used to identify the most likely number of species present in the samples using the ITS1 locus since this database included all sampled specimens. In the PTP model, speciation events are hypothesized according to the observed number of substitutions between sequences, using either a maximum likelihood (mPTP) or Bayesian solution (bPTP). The input Newick file was generated in FigTree from the BI phylogenetic tree used in the analysis described above. The algorithm was implemented on the website http://species.h-its.org/ptp/ with 10^5^ MCMC generations, 'burn-in' length of 10^4^ and a thinning of 10^2^. Convergence of the MCMC chains were visually inspected to confirm the equilibrium distribution.

### Scanning electron microscopy (SEM) analysis

To evaluate potential morphological differences in a novel identified *S. lupi* genotype, SEM analysis was performed to visualize the anterior and posterior extremities of four worms collected from dogs from Hungary. The worms were washed, fixed and dehydrated in an ethanol series as previously described [5]. Then, the worms were dried with a critical point dryer (Quorum K850, Quorum Technologies Ltd., Lewes, UK) with CO_2_, mounted on aluminum stubs, coated with iridium for 20 s (Quorum Spatter coater Q150T ES, Quorum Technologies Ltd.) and observed in a scanning electron microscope (JSM-IT100, JEOL USA Inc., Peabody, USA) in a 10 mm stage height operated at 3 kV. The obtained SEM images were compared to those from *S. lupi* and *S. vulpis* recently reported [5].

## Results

### Analysis of the *18S* gene

The *18S* gene (1611 bp) was successfully amplified from 87% (27/31) of the nematodes. The comparison of this locus showed nucleotide distances below 1% between *S. lupi* and *S. vulpis* (Additional file 3: Table S1). Nevertheless, three groups of genotypes (i.e. each containing identical sequences) were identified among the specimens, as follows: (i) *S. lupi* genotype 1 (*n* = 17) from dogs from Israel, South Africa and India; (ii) *S. lupi* genotype 2 (*n* = 4) from dogs only from Hungary; and (iii) *S. vulpis* (*n* = 6) from red foxes from Spain and Bosnia and Herzegovina specimens. Five polymorphic nucleotide sites were observed within the *S. lupi* genotypes 1 and 2, that included two indels, two nucleotide transitions (C to T and from A to G) and a transversion (G to C). The nucleotide distance between *S. lupi* genotype 1 and genotype 2 was 0.187%, while both *S. lupi* genotypes differed from *S. vulpis* by 0.062%. *Spirocerca lupi* genotypes 1 and 2 were 99% identical to a reference sequence of *S. lupi* (GenBank: AY751497.1). In addition, the distance between *S. lupi* genotype 1, *S. lupi* genotype 2 and *S. vulpis* to a *Spirocerca* sp. collected from the Island fox (*Urocyon littoralis*) from San Miguel Island in the USA ranged between 1.721–1.849%. Nucleotide pairwise distances of *Spirocerca* spp. analyzed herein with other members of the Spiruridae family ranged between 3.783–11.289% (Additional file 3: Table S1). An *18S* sequence from *S. lupi* genotype 2 was deposited in the GenBank database under the accession number MH628159.

**Fig. 1:**
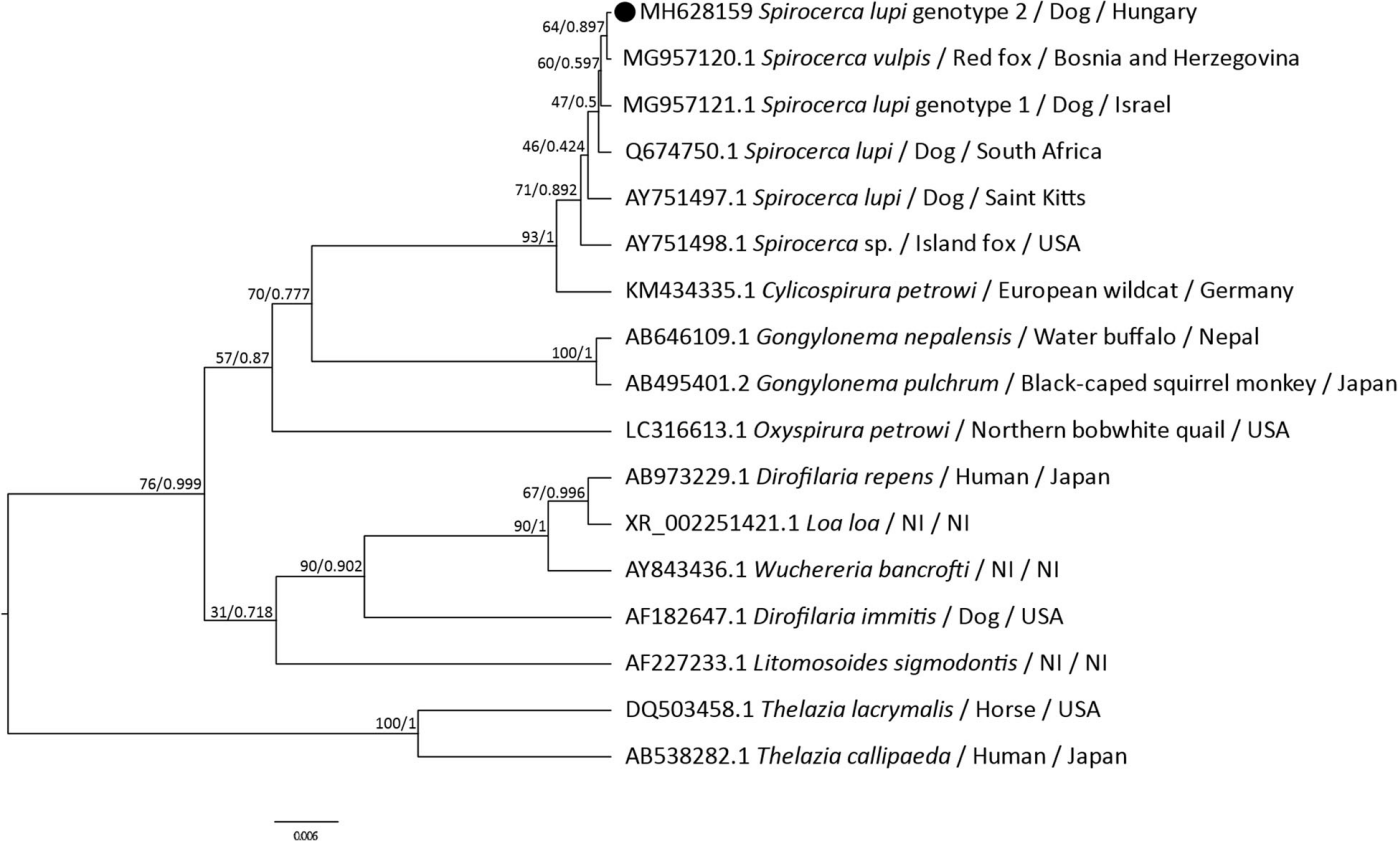
Phylogenetic trees for *Spirocerca* spp. based on the *18S* gene. Maximum likelihood and Bayesian trees inferred from a 1611 bp sequence of the *18S* gene of *Spirocerca* spp. and other species from the family Spiruridae. The sequence obtained in this study is indicated with a black circle. Bootstrap and posterior probability values, respectively, are shown above the branches

The ML and BI phylogenetic trees resulted in identical topologies (Fig. 1). Notably, both trees clustered all *Spirocerca* spp. together, with high bootstrap values (71%) and posterior probabilities (0.892). Nevertheless, *Spirocerca* spp. associated internal nodes were less supported, with the exception of the cluster of *S. vulpis* and *S. lupi* genotype 2 sequences (Fig. 1). Moreover, the separation of all *Spirocerca* spp. from other members of the family Spiruridae such as *C. petrowi*, *Gongylonema* spp. and *Oxyspirura petrowi* was evident, with high support values.

### Analysis of the ITS1 loci

Intra-individual variability in the ITS1 loci was detected in both *Spirocerca* spp. A total of 264 ITS1 sequences were obtained and analyzed from the 31 worms, i.e. 210 for *S. lupi* and 54 for *S. vulpis* (Table 3). Each individual nematode contained two or more different copies (up to 6 different copies per individual) of the ITS1 locus (hereinafter referred to as “haplotypes”), with sequence variations that ranged between 0.37–2.85% (Table 3). The worms with the smallest and largest median intra-individual variation were the *S. lupi* specimens N, O and P from India (with all sequences, except one, identical to each other) and the *S. lupi* specimen CA from Bosnia and Herzegovina (median = 1.47, 3rd interquartile range (IQR): 1.45–1.49), respectively. The intra-individual variation from *S. vulpis* specimens ranged between 0.37–1.80% (Table 3). The *S. lupi* sequences from Israel, South Africa, India and Australia were 96% identical to the *S. lupi* reference ITS1 sequence (GenBank: MF425539), described earlier [42]. Additionally, *S. lupi* sequences of worms collected from Hungarian and Italian dogs and *S. vulpis* were 72% and 64% identical to this same reference sequence.

**Table 3:** Intra-individual pairwise nucleotide distances (%) of the ITS1 sequences of *Spirocerca* spp.

Specimen	No. of sequences analyzed	Median (third interquartile range)	Minimal variation	Maximal variation
*S. lupi*				
A	10	0.37 (0.36–0.38)	0.37	0.71
B	10	0.37 (0.36–0.38)	0.37	0.71
C	9	0.73 (0.72–0.74)	0.37	1.42
D	11	0.73 (0.71–0.75)	0.37	2.85
E^a^	8	0.37 (0.30–0.44)	0.73	1.78
F	7	0.37 (0.30–0.44)	0.37	0.71
G	10	0.73 (0.72–0.74)	0.37	1.42
H	8	0.73 (0.72–0.74)	0.37	1.42
I	10	0.74 (0.73–0.75)	0.37	1.07
J	8	0.73 (0.723–0.80)	0.37	1.07
K	8	0.73 (0.72–0.74)	0.37	0.71
L	7	0.73 (0.71–0.75)	0.37	2.17
M	10	0.37 (0.36–0.38)	0.37	1.07
N^a^	9	0	–	0.71
O^a^	7	0	–	0.71
P^a^	8	0	–	0.71
Q	8	0.37 (0.36–0.38)	0.37	0.71
V	11	0.73 (0.72–0.74)	0.37	1.78
W	5	1.10 (1.08–1.12)	0.37	1.78
R	12	0.55 (0.54–0.56)	0.37	1.43
AA	10	0.74 (0.73–0.75)	0.37	1.79
BA	10	1.10 (1.08–1.12)	0.37	2.14
CA	8	1.47 (1.45–1.49)	0.37	1.43
DA	6	1.10 (1.08–1.12)	0.37	2.14
*S. vulpis*				
S	10	0.37 (0.36–0.38)	0.37	1.80
T^a^	3	0.37 (0.36–0.38)	0.37	0.72
U	7	0.73 (0.72–0.74)	0.37	1.42
X	11	0.37 (0.36–0.38)	0.37	1.42
EA	7	0.37 (0.36–0.38)	0.37	1.07
FA	6	0.37 (0.36–0.38)	0.37	0.72
GA	10	0.37 (0.36–0.38)	0.37	1.08

^a^All sequences of the specimen, except for one, were identical to each other

The G+C content and length of *S. lupi* ITS1 sequences differed between the different geographical locations (Table 4). First, pairwise comparisons showed significant differences in the length of the *S. lupi* sequences between most of the paired geographical locations (all *P* < 0.000001), except for South Africa and Australia, India and Australia, and, Hungary and Italy. In addition, the G+C content varied significantly between the worms from Israel and the other geographical locations (all *P* < 0.00001), and between India and the locations of Australia and Hungary (*P* = 0.00002 and 0.0005, respectively). In the case of *S. vulpis*, the G+C content and sequence lengths did not show significant differences between the sampling locations (all *P* > 0.05). Moreover, there were no differences when comparing the averages of these parameters between *S. vulpis* and *S. lupi* sequences (*P* = 0.031; not significant after correction for multiple comparisons).

**Table 4:** General characteristics of the ITS1 sequences of *Spirocerca* spp. according to the geographical location

Geographical location	G+C content (%) Mean ± SD	Length (bp) Mean ± SD	S	*h*	Hd ± SD	π ± SD
*S. lupi*						
Israel (*n* = 73)^a,b^	30.24 ± 0.82	398 ± 6	27	22	0.874 ± 0.026	0.0074 ± 0.0006
South Africa^a,b^ (*n* = 34)	31.56 ± 0.96	387 ± 12	9	8	0.886 ± 0.014	0.0064 ± 0.0008
India (*n* = 41)^b^	31.12 ± 1.02	380 ± 5	15	7	0.263 ± 0.049	0.0026 ± 0.0012
Australia (*n* = 16)^b^	31.95 ± 0.35	379 ± 2	9	9	0.825 ± 0.036	0.0051 ± 0.0010
Italy (*n* = 12)^c^	31.43 ± 0.70)	371 ± 2	5	6	0.758 ± 0.079	0.0050 ± 0.0014
Hungary (*n* = 34)^c^	31.79 ± 0.51	357 ± 8	11	10	0.722 ± 0.016	0.0055 ± 0.0027
Total (*n* = 210)	31.11 ± 1.05	383 ± 17	–	–	–	–
*S. vulpis*						
Spain (*n* = 20)^d^	31.33 ± 0.34	390 ± 3	5	6	0.445 ± 0.024	0.0022 ± 0.0020
Italy (*n* = 11)^d^	31.21 ± 0.47	391 ± 4	3	3	0.345 ± 0.107	0.0024 ± 0.0013
Bosnia and Herzegovina (*n* = 23)^d^	31.45 ± 0.30	390 ± 5	4	4	0.249 ± 0.020	0.0015 ± 0.0007
Total (*n* = 54)	31.36 ± 0.36	392 ± 4	–	–	–	–

^a^Contains one haplotype shared with Israeli and South African sequences

^b^Contains one haplotype shared by Israeli, South African, Indian and Australian sequences

^c^Contains one haplotype shared with Italian and Hungarian sequences

^d^Contains one haplotype shared with Spanish, Italian and Bosnian sequences

*Abbreviations*: n, number of obtained sequences; S, segregating sites; *h*, number of haplotypes; Hd, haplotype diversity; π, nucleotide diversity; bp, base pairs; SD, standard deviation

Five microsatellite motifs were found in the ITS1 sequences of both *Spirocerca* spp., namely (AA)_4_, (AT)_4_, (TA)_5_, (GT)_4,6_, (AC)_4–11_ and (TA)_4–6_. Of these, microsatellite (AC) was present in 88.6% (186/210) of the *S. lupi* ITS1 sequences and was found in four copies in the specimens from Israel, South Africa, India and Australia; and six to eleven copies in the worms from Hungary and Italy. In addition, microsatellite (TA)_5_ was observed in 100% (100/100) of the sequences of *S. vulpis* and *S. lupi* specimens from Hungary and Italy. Microsatellite (GT)_4,6_ was found in 89% (48/54) of the *S. vulpis* specimens.

Haplotype and nucleotide diversity parameters of the ITS1, Hd and π, respectively (Table 4), varied between geographical locations. The highest Hd was found in South Africa (0.886 ± 0.014) and Israel (0.874 ± 0.026), and the lowest in India (0.263 ± 0.049). Additionally, the sequences obtained from the worms from Israel had the highest π-value (0.0074 ± 0.0006), which was 28 times larger than the lowest π-value found in India (0.0026 ± 0.0012). In the case of *S. vulpis*, the Hd and π were highly similar between the collection sites and ranged between 0.249–0.445 and 0.0015–0.0024, respectively.

**Fig. 2:**
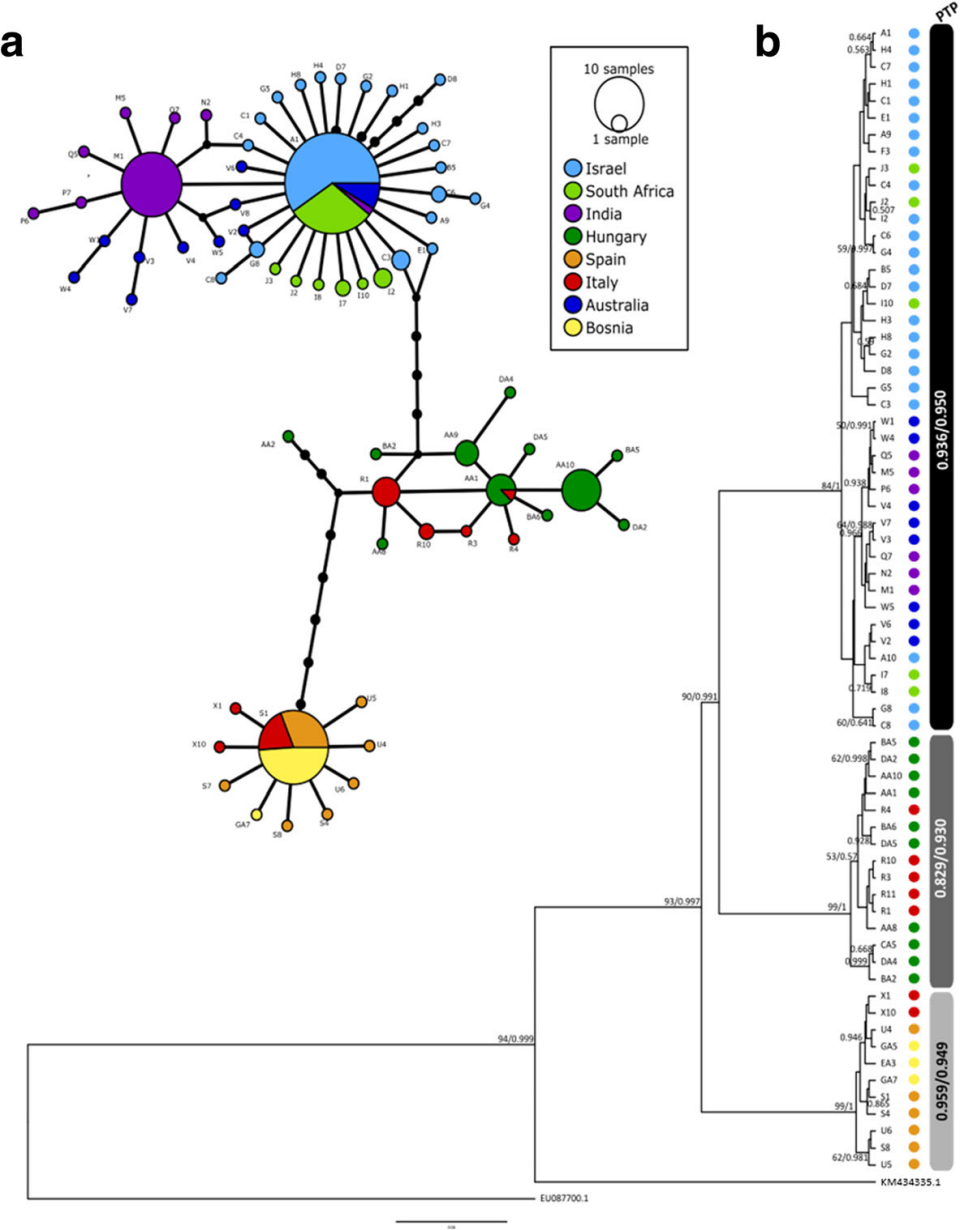
Analysis of the ITS1 loci of *Spirocerca* spp. **a** Templeton-Crandall-Sing (TCS) haplotype network of the ITS1 sequences of *Spirocerca* spp. Each colored circle represents a haplotype; circle size is proportional to the number of sequences sharing the same haplotype. The black circles denote hypothetical sequences connecting each haplotype. **b** Bayesian inference tree for the ITS1 haplotypes of *Spirocerca* spp. Bootstrap (BS) and posterior probability (PP) values, respectively, are shown above branches. Only BS and PP values above 50% or 0.5, respectively, are shown. *Cylicospirura petrowi* (KM434335.1) and *Dirofilaria immitis* (EU087700.1) sequences were used as the outgroups. The results of the Poisson tree processes (PTP) analysis are summarized to the right of the taxa names as grey scale bars with the support partition values found by the maximum likelihood and Bayesian solutions. The identity of each taxon in the tree and network is color-coded according to its geographical location

Overall, 62 and 13 ITS1 haplotypes were obtained from the *S. lupi* and *S. vulpis* nematodes, respectively (Table 4). In *S. lupi*, 93% (54/57) of these haplotypes were unique to a specific geographical origin, as depicted in the TCS network (Fig. 2a). However, three haplotypes were shared between worms from different geographical populations: one haplotype was found in Israeli and South African specimens; one haplotype in Israeli, South African, Indian and Australian worms; and one haplotype in Hungarian and Italian specimens. In *S. vulpis*, only one haplotype was shared between the nematodes from Spain, Italy and Bosnia and Herzegovina. The ML and BI trees of the ITS1 inferred two distant genetic clades, each containing only *S. lupi* or *S. vulpis* haplotypes, with high support values (bootstrap and posterior probability values of 93 and 0.997, respectively; Fig. 2b). The *S. lupi* haplotypes were sub-divided into two major genetic clades, one including Israeli, South African, Indian and Australian haplotypes, and another one including Hungarian and Italian haplotypes, with high support values (bootstrap and posterior probability values of 90 and 0.991, respectively; Fig. 2b). ITS1 haplotype sequences were deposited in the GenBank database under the accession numbers MH630178-MH630235 for *S. lupi* and MH630236-MH630246 for *S. vulpis.*

Despite the large ITS1 intra and inter-individual variability detected, a clear differentiation between the *S. lupi* and *S. vulpis* specimens was evident by the PCoA plot generated with Nei’s genetic distance, which mirrored the clustering obtained from the *18S* analysis (i.e. two S. *lupi* genotypes and one *S. vulpis* genotype; Additional file 4: Figure S3a). Moreover, when this analysis was performed including only the *S. lupi* sequences, a subdivision of genotype 1 specimens was obtained, resulting in three main clusters (Additional file 4: Figure S3b), as follows: (i) sequences from Israeli and South African specimens (genotype 1A); (ii) sequences from Indian and Australian specimens (genotype 1B); and (iii) sequences from Italian and Hungarian specimens (genotype 2). The percentage of variation explained by the axes corresponded to 88.3%, separating both genotypes 1 from genotype 2, and 6.6%, separating genotypes 1A and 1B.

The pairwise nucleotide distances revealed a low variation within each *S. lupi* genotype and *S. vulpis*, and a high variation when comparing between these groups*.* When the ITS1 sequences were compared within each group (i.e. intra-genotypic variation), the median nucleotide distances were 0.73% for *S. lupi* genotypes 1A, ranged between 0.10–0.73% for *S. lupi* genotypes 1B, 0.55–1.10% for *S. lupi* genotype 2, and 0.37–0.74% for *S. vulpis*. The median *S. lupi* inter-genotypic variation ranged between 1.10–9.33%. Genotypes 1A and 1B were the closest with low inter-genotypic variation (median = 1.10, 3rd IQR: 1.08–1.12), and the more distantly related were genotypes 1B and 2 (median = 8.06, 3rd IQR: 7.98–8.12). Furthermore, the nucleotide distance between the *S. lupi* genotypes and *S. vulpis* ranged between 6.58–9.93%.

Finally, the Poisson tree processes (PTP) analysis determined three putative species in the complete *Spirocerca* spp. database according to the Maximum Likelihood and Bayesian solutions (observed in Fig. 2b as grayscale bars): (i) *S. lupi* genotype 1 (support > 0.936); (ii) *S. lupi* genotype 2 (support > 0.829); and (iii) *S. vulpis* (support > 0.949). This analysis did not support a division within genotype 1 of *S. lupi*.

### Analysis of the *cox*1 gene

Two different PCRs were performed to obtain a larger sequence of the *cox*1 gene (Additional file 1: Figure S1). Twenty-six of the tested 31 specimens gave positive results using both reactions. However, the *cox*1 gene from one *S. lupi* worm from Hungary, one from Israel and none of the worms from Australia and Italy, could be amplified by both reactions.

The analysis of the *S. lupi cox*1 sequences from fragment A resulted in a clustering of the specimens with the same pattern of organization as in the *18S* and ITS1 analyses. Accordingly, Israeli, South African and Indian *S. lupi* worms grouped together (i.e. genotype 1), and apart from the Hungarian nematodes (i.e. genotype 2) and *S. vulpis*. Overall, 16 *cox*1 haplotypes were obtained from the *S. lupi* specimens and five from *S. vulpis* specimens. All Israeli and South African sequences had a length of approximately 1000 bp, 400 bp larger than the expected amplicon [9] (Additional file 2: Figure S2a). The larger PCR product was a result of a 3 bp nucleotide substitution in the targeted NTF primer sequence and the presence of an alternative 300 bp upstream NTF region. The latter was confirmed by an alternative PCR assay using primers NTF and NTInt (Additional file 1: Figure S1, Additional file 2: Figure S2b). The pairwise nucleotide distances within *S. lupi* genotypes 1 and 2 and *S. vulpis* were lower (2.18 ± 1.04%, 0.18% and 1.06 ± 0.46%, respectively) than when comparing between these groups (from 5.31 to 9.34%) (Additional file 5: Table S2). The *cox*1 sequences from the *S. lupi* specimens of genotype 1 were 97 to 99% identical to the *cox*1 sequence of *S. lupi* complete mitochondrial genome deposited in GenBank from China (KC305876.1), while *S. lupi* genotype 2 sequences were 94% identical to this reference sequence. Particularly, this *S. lupi* reference sequence was more similar to the haplotypes obtained from Indian specimens (distance of 0.37–0.55%) than to the *S. lupi* genotype 2 sequences (distance of 6.23–6.42%). In addition, the distances between all our *Spirocerca* spp. to other *Cylicospirura* spp. ranged between 9.52–12.27%.

The *cox*1 sequences from amplified fragment B were classified in eleven haplotypes from the *S. lupi* specimens and three from *S. vulpis* specimens. These haplotypes grouped virtually with the same organization as obtained with the *cox*1 fragment A. A higher nucleotide distance was obtained when *S. lupi* genotype 1 sequences were compared to genotype 2 (7.25 ± 0.73%) and *S. vulpis* (7.39 ± 0.87%) (Additional file 6: Table S3). Furthermore, the nucleotide distance between *S. lupi* genotype 2 and *S. vulpis* was 7.76 ± 0.39 (7.23–8.38%). The *S. lupi* haplotypes showed a high similarity to the *S. lupi* reference sequences from South Africa [2] and Peru [11] (Additional file 6: Table S3). The *S. lupi* haplotypes of genotype 1 were 3.05 ± 1.76 and 3.02 ± 1.50% different to the sequences of South African worms obtained from dogs and black-backed jackals (GenBank: KY495493.1-KY495505.1) [2] and from the Andean fox in Peru (GenBank: KY634868.1-KY634870.1) [11], respectively. Moreover, the sequences from the South African worms obtained in this study (with the exception of specimen L) were highly similar (average pairwise nucleotide distance: 0.67 ± 0.28%) to the sequences previously characterized from this country by Rothmann & de Waal [2]. The South African specimen L was more closely related to *S. lupi* from Israel than to the other South African specimens. In addition, the *S. lupi* worms from Israel showed the highest similarity to the Peruvian *S. lupi* sequences (average pairwise nucleotide distance: 1.40 ± 0.53%) (Additional file 6: Table S3).

**Fig. 3:**
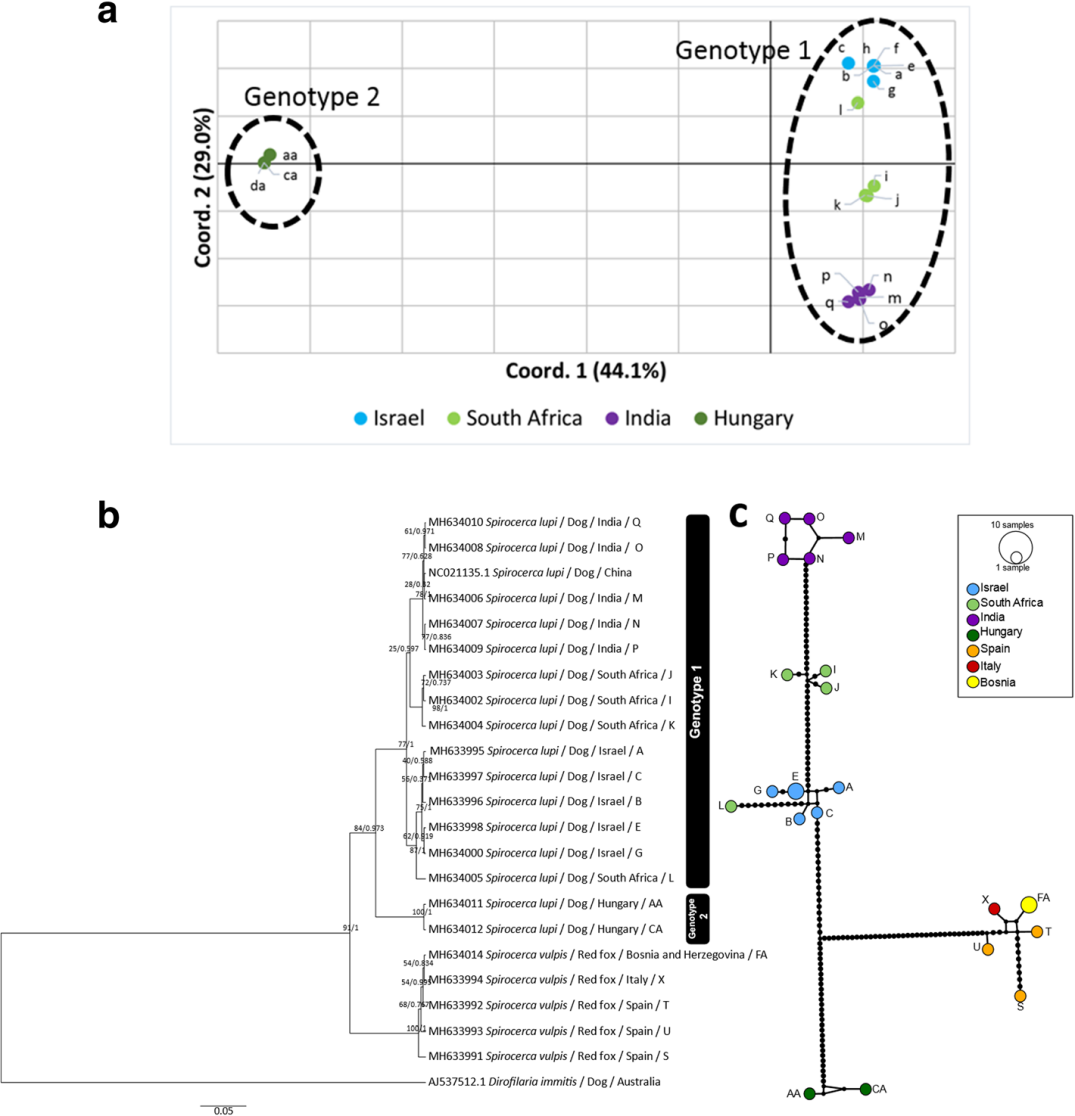
Analysis of the *cox*1 combined fragment of *Spirocerca* spp. **a** A principal coordinate analysis (PCoA) scatter plot showing Nei’s genetic distances of the *cox*1 combined fragment sequences of *S. lupi*. **b** Bayesian inference tree of the *cox*1 combined fragment (317 to 1201 bp) of *Spirocerca* spp. Bootstrap and posterior probability values are shown above branches. **c** Haplotype network of the *cox*1 sequences. Each circle is colored according to the geographical origin of the haplotype; circle size is proportional to the number of sequences sharing the same haplotype. The black circles represent the number of mutations between the haplotypes

The concatenated *cox*1 sequence (fragments A and B) replicated the grouping obtained by the single *cox*1 fragments (Fig. 3a-c). Sixteen haplotypes were found in *S. lupi* and five in *S. vulpis* (Table 5). There were only two shared haplotypes in the *S. lupi* specimens, one between three Israeli specimens and another between two Hungarian worms. The Hd was 1.00 (i.e. all sequences were different) in the nematodes obtained from *S. lupi* from South Africa and India and *S. vulpis* from Spain. Moreover, since all *cox*1 sequences of worms from Bosnia and Herzegovina were identical, only one haplotype was found, and the diversity parameters were equal to 0. Additionally, the highest values of π were obtained from the nematodes collected from South Africa (0.0175 ± 0.0075) and Spain (0.0149 ± 0.0043) (Table 5). As obtained for the separate *cox*1 fragments, the nucleotide pairwise distances were higher when comparing between the haplotypes of *S. lupi* genotypes and *S. vulpis* (5.91–8.63%) than the distance within each group (0.12–3.55%) (Additional file 7: Table S4, Fig. 3a). The BI and ML trees (Fig. 3b) separated with high confidence both *S. lupi* genotypes (posterior probability of 0.973 and bootstrap support of 84). Two subgroups were obtained inside the *S. lupi* genotype 1: (i) Israeli sequences and one specimen from South Africa; and (ii) the remaining sequences from South Africa and India. The *S. lupi* genotypes 1 and 2 were 96–99% and 93% identical, to the mitochondrial genome of *S. lupi* from China (GenBank: KC305876.1), respectively. In addition, the *S. lupi* reference sequence from China was located next to the Indian sequences. These same observations were replicated in the haplotype network (Fig. 3c). However, the latter analysis showed an additional separation between Israeli, South African (except for one sequence which was more related to Israeli sequences) and Indian haplotypes. The newly generated sequences were deposited in the GenBank database under the accession numbers MH633995-MH634013 (*Spirocerca lupi*), and MH633991-MH633994 and MH634014-MH634016 (*S. vulpis*).

**Table 5:** Genetic diversity of the *cox*1 of *Spirocerca* spp. obtained from different geographical locations

Geographical location	S	*h*	Hd ± SD	π ± SD
*S. lupi*				
Israel (*n* = 7)	9	5	0.857 ± 0.137	0.0042 ± 0.0009
South Africa (*n* = 4)	19	4	1.000 ± 0.177	0.0177 ± 0.0067
India (*n* = 5)	4	5	1.000 ± 0.126	0.0026 ± 0.0004
Hungary (*n* = 3)	1	2	0.667 ± 0.099	0.0024 ± 0.0011
*S. vulpis*				
Spain (*n* = 3)	14	3	1.000 ± 0.272	0.0110 ± 0.0034
Bosnia and Herzegovina (*n* = 3)	0	1	0	0

*Abbreviations*: n, number of obtained sequences; S, segregating sites; *h*, number of haplotypes; Hd, haplotype diversity; π, nucleotide diversity; SD, standard deviation

### SEM of specimens from Hungary

**Fig. 4:**
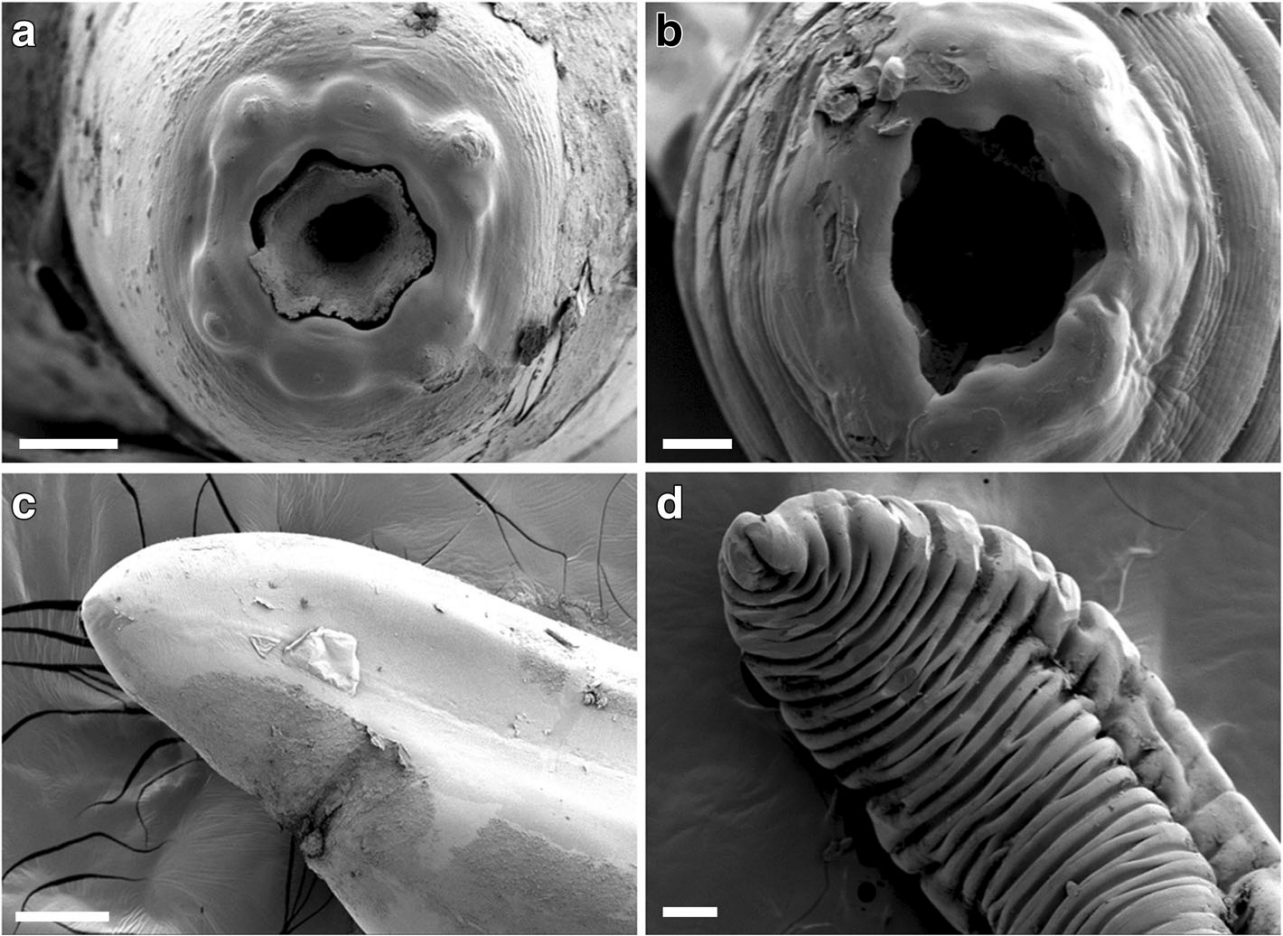
Scanning electron microscopy analysis on *S. lupi* specimens collected in Hungary. **a**, **b** Anterior part of the worm showing four cephalic papillae, one pair of amphids and a hexagonal oral opening. **c** Anal opening observed in the posterior extremity of a female. **d** Posterior extremity of a juvenile male. *Scale-bars*: **a**, 50 μm; **b**, 20 μm; **c**, **d**, 100 μm

The four specimens (two females and two males) collected from Hungary (i.e. *S. lupi* genotype 2) exhibited four cephalic papillae and a pair of amphids in the anterior extremity by SEM analysis. The buccal capsule did not show any teeth-like or other sclerotized structures (Fig. 4a, b), the main morphological characteristic that allows the differentiation between *S. lupi* (absent) and *S. vulpis* (present) specimens [5]. Additionally, the posterior extremity of females showed morphological similarities to both *S. lupi* and *S. vulpis* specimens (Fig. 4c). Eggs could be distinguished in different cross-sections of the worms (Additional file 8: Figure S4). The posterior extremity of both males was ventrally curved and resembled juvenile stages since neither the cloaca, spiculae, pre-anal nor post-anal papillae were evident (Fig. 4d). Furthermore, the parallel longitudinal ridges, characteristic of *Spirocerca* spp. males, were not observed. Instead, broad bands of approximately 30 μm apart with cuticular striations separated approximately by 3 μm were distinguished.

## Discussion

The present study explored the phylogenetic relationships and genetic variation among *S. lupi* and *S. vulpis* collected from eight different geographical locations of four continents*.* With the use of ribosomal (*18S*, ITS1) and mitochondrial (*cox*1) genetic targets, different *S. lupi* genotypes were classified and associated with geographical distributions. Moreover, this study revealed a high intra-individual variation of the ITS1 locus in all specimens from both *Spirocerca* spp.

The *S. lupi* specimens were clearly separated into different genotypes according to the sampling location: genotype 1 found in specimens from Israel, South Africa, India and Australia; and genotype 2 represented by specimens from Hungary and Italy. These findings were supported by the analysis of the highly diverse ITS1 and *cox*1 loci, and the more conserved *18S* gene. Within *S. lupi* specimens, the ITS1 variation ranged between 0.32–8.54%, when comparing specimens from genotype 1 with genotype 2. A lower intra-specific variation (i.e. 0.3–2.5%) has been described in ITS1 fragments from *Thelazia* spp. [18], and *Trichuris* spp. (0.2–0.7%) [43]. The high variation presented herein supports the presence of two clearly different genotypes, since the nucleotide distances decrease from 8.54% to 2.50% when analyzing these percentages separately according to the genotype. Moreover, the specimens from genotype 2 were classified as putative species by PTP analyses (support: 0.829 and 0.930). Thus, genotype 2 might be considered an intermediate entity between *S. lupi* genotype 1 and *S. vulpis*, as demonstrated by pairwise nucleotide distances, phylogenetic trees, haplotype networks, and the sharing of microsatellites with *S. lupi* genotype 1 and *S. vulpis*. The genetic distance between both *S. lupi* genotypes can be explained by small breeding population sizes, low infection prevalence within regions, or due to the high specificity of *S. lupi* to its definitive or intermediate hosts in each geographical location [14]. The prevalence of infection in a geographical area affects the genetic diversity of the parasite, since parasites in low prevalence regions will hardly find other genotypes reducing opportunities for sexual recombination. A previous study on *S. lupi* showed that even in geographical areas separated by 100 km, there was lack of panmixia, probably as a result of clumped transmission of infra-populations [12]. The latter will lead to a genetic differentiation of populations from geographically separated locations after a long period of time [44], which may eventually result in allopatric speciation [14]. The large genetic difference between the *S. lupi* genotypes, suggested that the specimens collected from Hungarian and Italian dogs (i.e. genotype 2) could be a separate (i.e. specimens with genetic and morphological differences) or cryptic species (i.e. specimens with genetic differences but morphologically identical) [45]. However, the SEM analysis did not show evidence of morphological differences between genotype 2 specimens and *S. lupi* from Israel (i.e. genotype 1) [5], suggesting that this genotype could represent a cryptic species. Nevertheless, the morphological analysis of a larger number of sexually mature specimens collected from dogs from Hungary, Italy or Bosnia and Herzegovina or other locations in Europe is required to confirm this taxonomical status.

The ITS1 loci of *Spirocerca* spp. were characterized for the first time and a high genetic intra-individual variation was detected. We found that each nematode had different copies of the ITS1, with up to six different haplotypes observed per worm specimen. Intra-individual variation in the ITS1 has also been reported in *Trichuris* spp. [43], *Echinococcus granulosus* [20] and *Paragonimus westermani* [21]. The ITS regions are important for maintaining the secondary structure of rRNA genes and for the processing of transcribed rDNA [46]. Highly repeated sequences, such as rDNA, are maintained homogeneously within individuals and populations by concerted evolution, which assumes the continual turnover of repeats by recombination mechanisms [47]. However, sequence variation may exceed the homogenization process due to interbreeding of siblings or reduced genetic recombination [21]. Interestingly, despite the high variation found within each nematode, the ITS1 phylogenetic analyses clearly separated both *S. lupi* genotypes and differentiated them from *S. vulpis*. Furthermore, the variation of the ITS1 showed an additional division of the *S. lupi* genotype 1 into two groups, one containing Israeli and South African specimens and the other containing Indian and Australian ones.

South African and Israeli specimens were classified together as genotype 1A according to the ITS1 database. This finding suggests potential panmixia among populations of these two locations and highlights the possible migration of *S. lupi-*infected hosts, either definitive, intermediate or paratenic, from Africa to the Middle East or *vice versa*. Migration is an important evolutionary mechanism that allows subpopulations to share genetic information and contributes to adaptation to local environments [44, 48]. Indeed, the first cases of a spirocercosis outbreak in Israel were detected in the late 1980’s and early 1990’s in Ramat Gan, central Israel, in parasitized dogs that frequented the surroundings of a safari park that housed animals brought directly from Africa [49]. This may have been the source of this outbreak and may also explain the ITS1 genetic similarity in *S. lupi* populations between Israel and Africa. However, only one South African haplotype was placed next to the Israeli sequences in the *cox*1 analysis, which might be explained by the different inheritance mechanisms of *cox*1 and ITS1 and the genetic turnover of these loci [45]. Surprisingly, *S. lupi* Israeli *cox*1 sequences were highly similar to a set of *S. lupi* obtained from the Andean fox (*Lycalopex culpaeus*) in Peru [11]. A high genetic similarity among *S. lupi* collected from two different canid hosts has been previously observed [2], with worms obtained from dogs and black-backed jackals in South Africa sharing two haplotypes, and thus suggesting the transmission between those canid species that live in sympatry. Moreover, our analysis shows that high similarity between specimens can be evident even when collected from two canids that are not in the same geographical area. This situation might also reflect the migration of infected hosts between both geographical locations [44].

The *cox*1 locus showed a high genetic interspecific variation and diversity, as demonstrated by the nucleotide differences between *S. lupi* genotypes and *S. vulpis* (0.17–11.07%) and the haplotype diversity within each species. For this gene, almost every nematode specimen contributed with one sequence variant, and only three worms from Israel and two from Hungary shared the same variant. Previous studies have addressed the variability in the *cox*1 gene of *S. lupi*. For instance, Traversa et al. [9] reported two haplotypes in 20 adult worms collected from undetermined hosts, one haplotype from specimens from Israel, Iran and South Africa, and another from Austrian and Italian specimens, with only six nucleotide substitutions separating them [9]; these findings correlate to genotypes 1 and 2 found in this study. However, we found a greater variation, with 19 haplotypes in total and 89 subtitutions within all *S. lupi* specimens, of which 30 contributed to the separation of the two genotypes. In addition, there were subgroups within each genotype according to geographical areas, since low *cox*1 pairwise nucleotide distances were found among the worms from the same country (0.263–0.827%), and higher distances were detected when comparing between locations (1.418–3.546%). Furthermore, a previous study of *S. lupi* conducted in 60 worms collected from 20 dogs confined to 300 km^2^ in South Africa found 11 haplotypes and a low genetic differentiation using the *cox*1 locus [10], which supports our results of low diversity within geographical locations. Finally, Rothmann & de Waal [2] reported 11 haplotypes in 49 nematodes from 31 dogs from South Africa. The difference in the number of haplotypes found in the latter study and ours might rely on the algorithms used for haplotype phasing, of which some are more strict than others [50]. Another possibility may be due to the sampling of sibling worms which tend to inbreed, reducing the genetic variability [14]. However, in our case, even sibling worms had different haplotypes, which highlights the large genetic variability within the studied nematodes.

Studies on the genetic diversity and differentiation between parasites are of special importance since they can expand the understanding of their distribution patterns and processes of speciation [14]. There is still limited information regarding the evolutionary history and speciation processes in *S. lupi* and *S. vulpis*, contrary to knowledge on other helminth parasites, such as *Fasciola hepatica* [8], *Fascioloides magna* [51] and *Anisakis* spp. [52]. For instance, a high genetic diversity within each host but low differentiation between populations was found in *F. hepatica*, with the implications of possible spread of anthelmintic resistance genes. Additionally, a migratory route of hosts infected with *F. magna* and a speciation foci was found in a genetic population study [51]. *Spirocerca* spp. have been suggested as parasites of domestic dogs that have spilled over to wildlife [53]. The latter assumption has been based on reports of *S. lupi-*like life stages found in other canid species [54–58]. However, most of these reports lack detailed morphological and genotypic screening, thus, it cannot be ruled out that the observed nematodes may represent different *S. lupi* genotypes or different *Spirocerca* spp. For instance, morphometric and phylogenetic analyses have led to the description of *S. vulpis* collected from red foxes, which had been previously misclassified as *S. lupi*, based on macroscopic and microscopic observations of the worms [59]. Therefore, many questions regarding the speciation and evolutionary history of *Spirocerca* spp. rise with our results, including if this genus originated in fact from dogs and spread to wildlife animals, or the spread of this infection occurred in the opposite direction.

## Conclusions

A high genetic variability of *Spirocerca* spp. was found in specimens from Asia, Africa, Europe, and Oceania based on the analysis of the ITS1 and *cox*1 loci. This included a high degree of variation in the ITS1 loci with up to six haplotypes in a single worm and marked polymorphism in the *cox*1 gene of nematodes from different origins. The high genetic diversity found herein led to the identification of two *S. lupi* genotypes which have different geographical distributions. This study suggests that migration and low prevalence in each geographical location studied may have been the driving forces of diversification with the genus *Spirocerca*.

## Additional files

Additional file 1: **Figure S1.** Diagram of primers employed for the amplification of different fragments of the *18S* (**a**), ITS1 (**b**) and *cox*1 (**c**) loci. PCR with primers NTF and NTInt was used for the confirmation of a 300 bp upstream NTF binding regions in South African and Israeli specimens. All amplicons obtained in the PCRs were sequenced. (TIF 397 kb)

Additional file 2: **Figure S2.**Agarose gels stained with ethidium bromide showing the ~600 and 1000 bp amplicons obtained during the amplification of the *cox*1 fragment A using the NTF and NTR primers (**a**) and the ~300 bp amplicons after running PCR using the NTF and NTInt primers (**b**). (TIF 1837 kb)

Additional file 3: **Table S1.** Pairwise nucleotide distance of the *18S* gene (1611 bp fragment) expressed as percentages between specimens of *Spirocerca* spp. and other nematodes of the order Spirurida. (DOCX 18 kb)

Additional file 4: **Figure S3.** Principal coordinate analysis (PCoA) scatter plot showing Nei’s genetic distances of the ITS1 sequences of *S. lupi* (**a**) and *Spirocerca* spp. (**b**) according to the sampling locations. The percentage of total variation attributed to each axis is indicated next to each coordinate. Each specimen is represented as a color-coded circle according to the geographical origin. (TIF 385 kb)

Additional file 5: **Table S2.** Pairwise nucleotide differences (%) between *cox*1 fragment A (317 to 967 bp) haplotypes obtained from *S. lupi* and *S. vulpis*, and reference *cox*1 sequences of *Spirocerca* spp. and *Cylicospirura* spp. available on GenBank. (DOCX 22 kb)

Additional file 6: **Table S3.**Pairwise nucleotide differences (%) between *cox*1 fragment B (807 to 1201 bp) haplotypes obtained from *S. lupi* and *S. vulpis*, and *cox*1 reference sequences of *S. lupi* and *D. immitis* available on GenBank. (DOCX 31 kb)

Additional file 7: **Table S4.**Pairwise nucleotide distance (%) in the *cox*1 (317 to 1201 bp) haplotypes of *Spirocerca* spp. (DOCX 16 kb)

Additional file 8: **Figure S4.** Scanning electron microscopy of a *S. lupi* female from Hungary showing the eggs in a uterus cross-section. (TIF 2174 kb)

## Abbreviations

AIC: Akaike information criterion
BI: Bayesian inference
bPTP: Bayesian solution for the Poisson tree processes
*cox*1: Cytochrome *c* oxidase subunit 1
ESS: Effective sample size
*h*: Number of haplotypes
Hd: Haplotype diversity
HKY: Hasegawa-Kishino-Yano
ITS1: Internal transcribed spacer 1
MCMC: Markov chain Monte Carlo
ML: Maximum-likelihood
mPTP: Maximum likelihood for the Poisson Tree Processes
PCR: Polymerase-chain reaction
PTP: Poisson Tree Processes
rDNA: Ribosomal DNA
S: Segregating sites
SEM: Scanning electron microscopy
TCS: Templeton-Crandall-Sing haplotype network
TIM: Transitional model
TPM: Three parameter model
UPW: Ultrapure water
*18S*: Small ribosomal subunit
π: Nucleotide diversity
