# Supplementary figures and images for: Phylogenetic analysis of Spirocerca lupi and Spirocerca vulpis reveal high genetic diversity and intra-individual variation
Source: Parasit Vectors. 2018 Dec 14;11:639. doi: 10.1186/s13071-018-3202-0 (PMC6295112; doi:10.1186/s13071-018-3202-0)

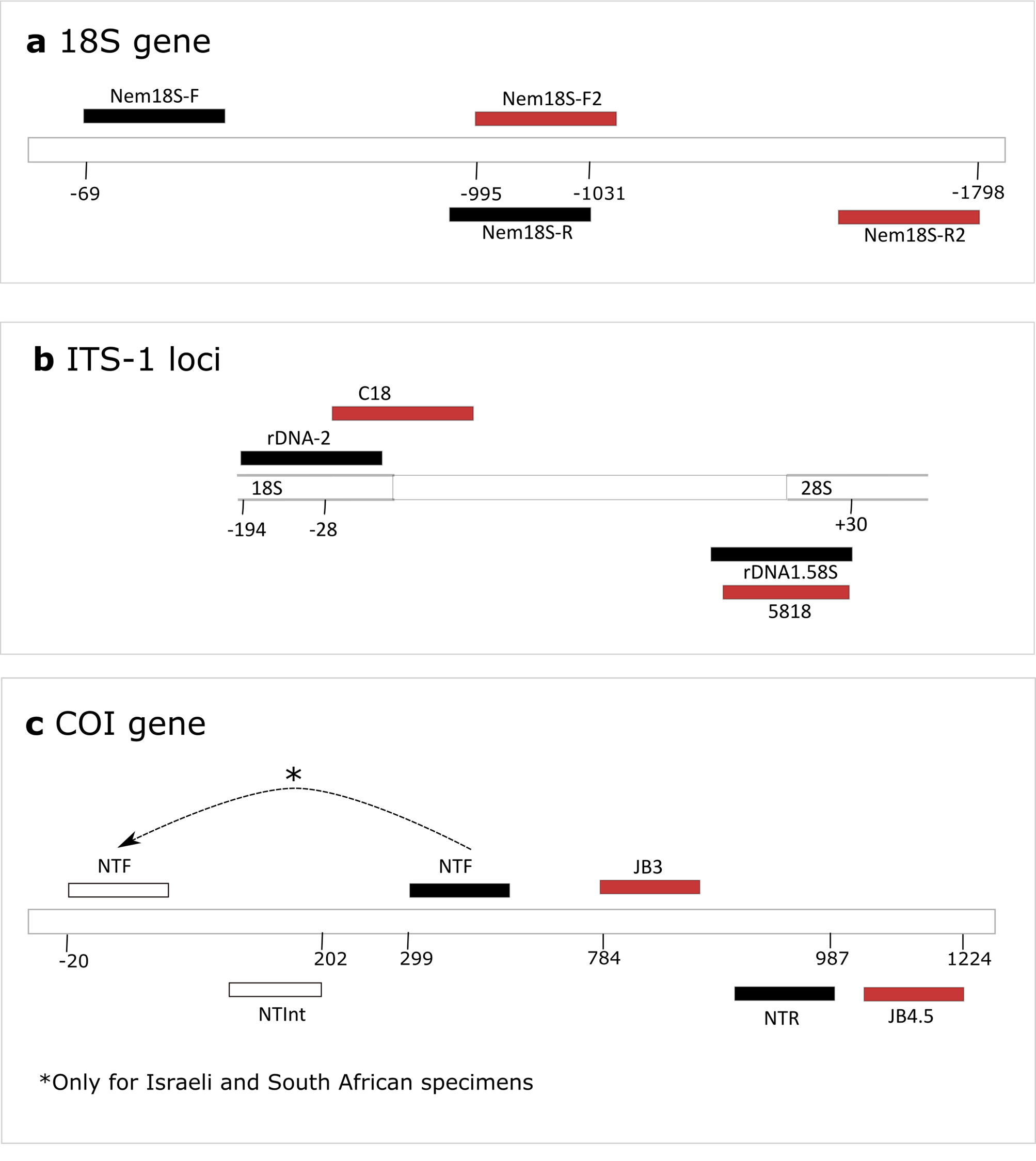

Supplement: Supplementary file 1 — Figure S1. Diagram of primers employed for the amplification of different fragments of the 18S (a), ITS1 (b) and cox1 (c) loci. PCR with primers NTF and NTInt was used for the confirmation of a 300 bp upstream NTF binding regions in South African and Israeli specimens. All amplicons obtained in the PCRs were sequenced. (TIF 397 kb) [file 13071_2018_3202_MOESM1_ESM.tif]

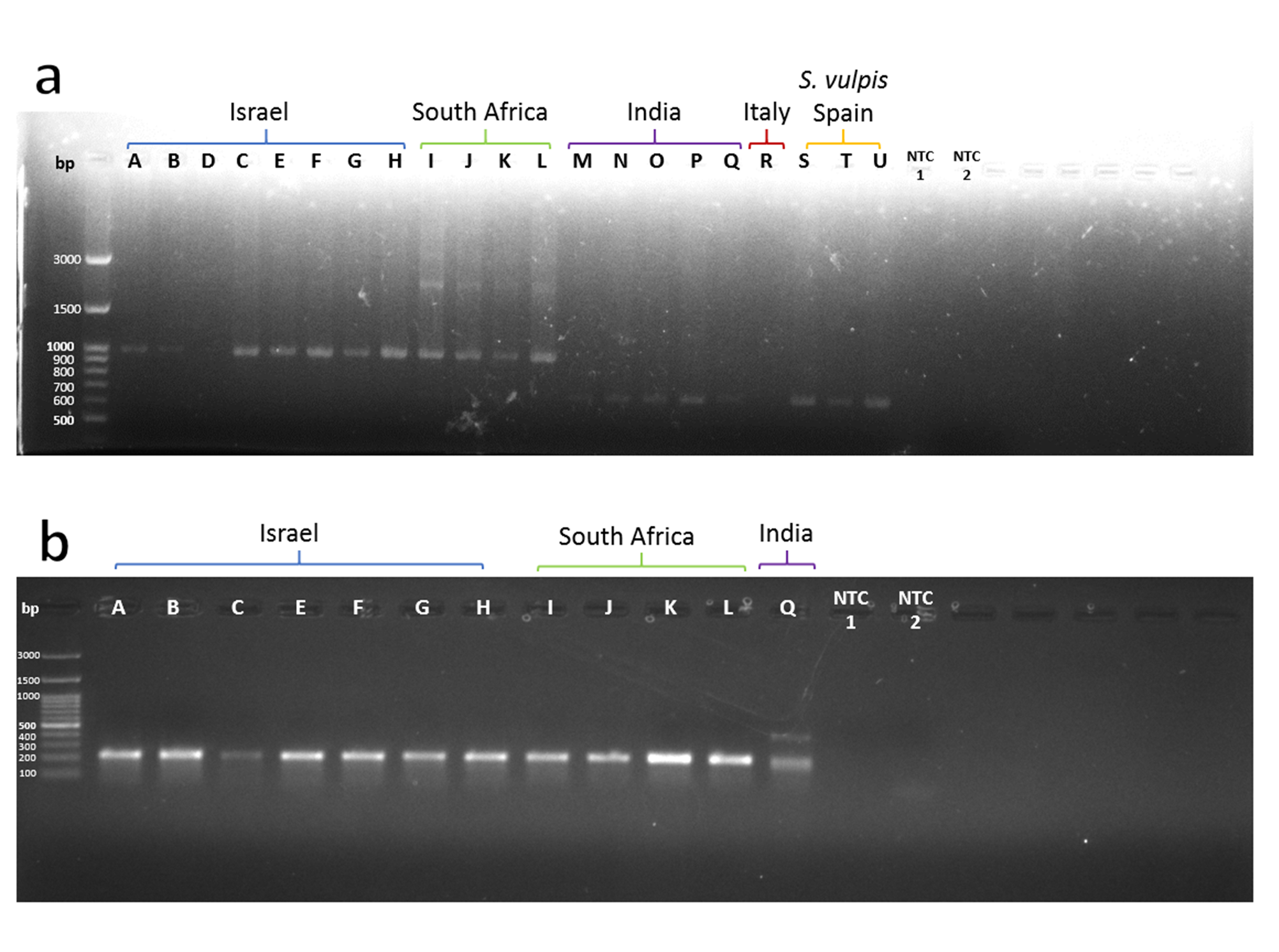

Supplement: Supplementary file 2 — Figure S2.Agarose gels stained with ethidium bromide showing the ~600 and 1000 bp amplicons obtained during the amplification of the cox1 fragment A using the NTF and NTR primers (a) and the ~300 bp amplicons after running PCR using the NTF and NTInt primers (b). (TIF 1837 kb) [file 13071_2018_3202_MOESM2_ESM.tif]

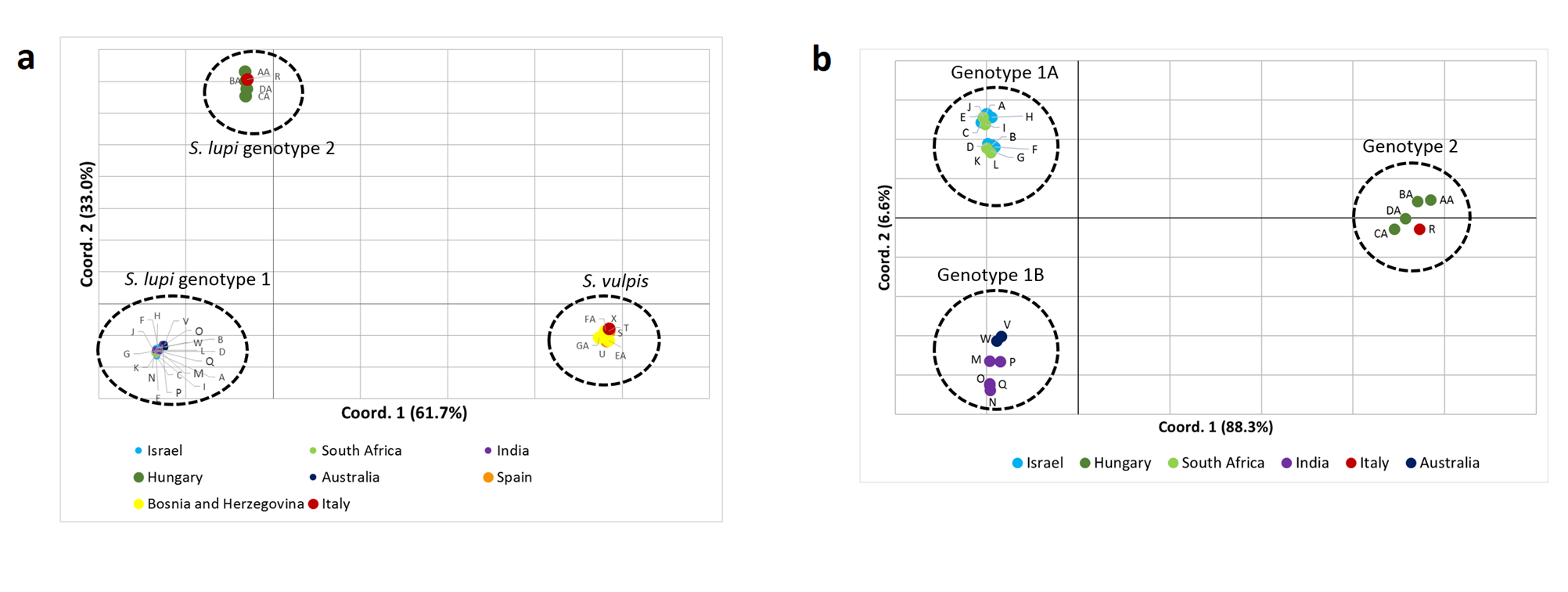

Supplement: Supplementary file 4 — Figure S3. Principal coordinate analysis (PCoA) scatter plot showing Nei’s genetic distances of the ITS1 sequences of S. lupi (a) and Spirocerca spp. (b) according to the sampling locations. The percentage of total variation attributed to each axis is indicated next to each coordinate. Each specimen is represented as a color-coded circle according to the geographical origin. (TIF 385 kb) [file 13071_2018_3202_MOESM4_ESM.tif]

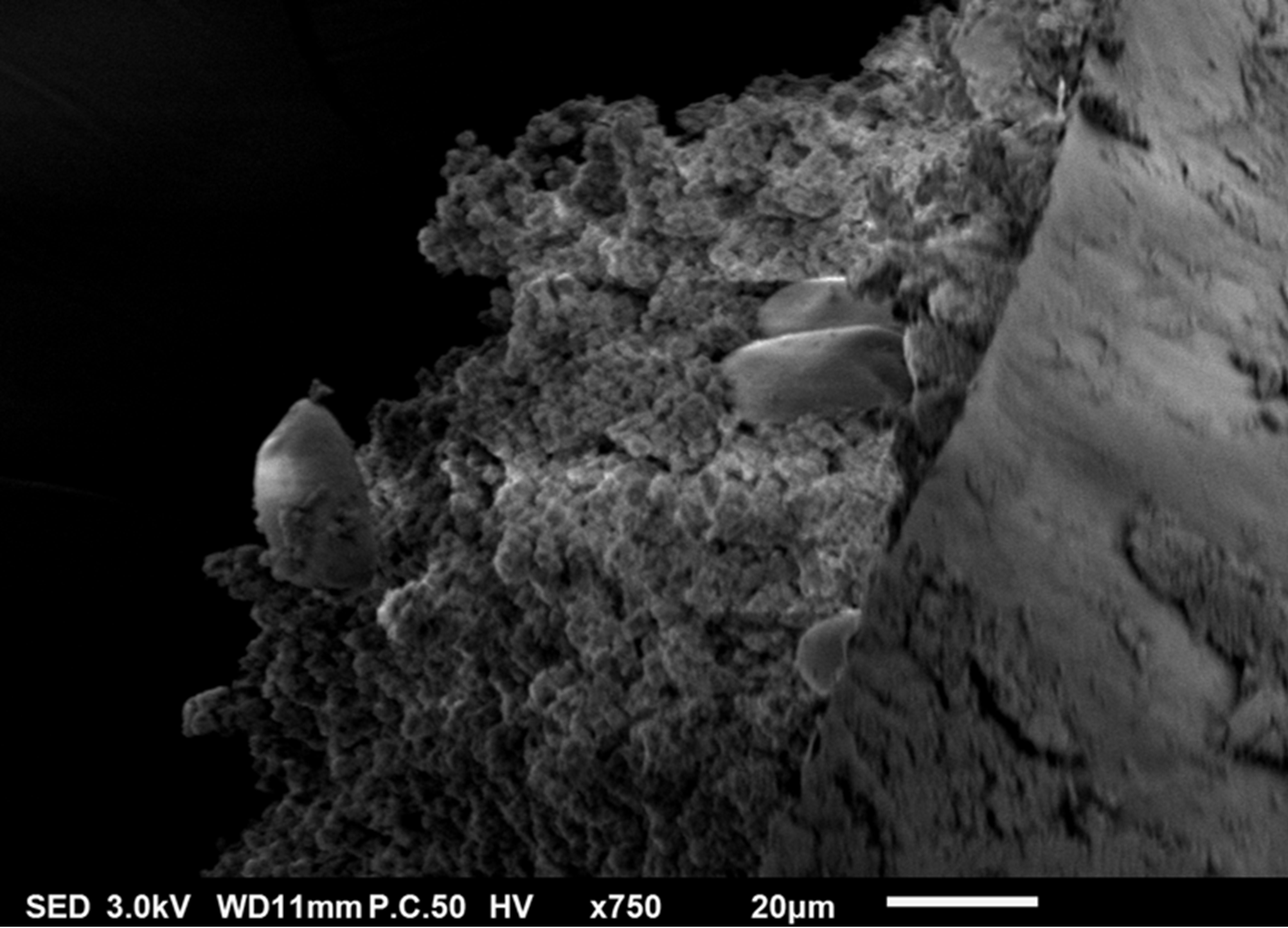

Supplement: Supplementary file 8 — Figure S4. Scanning electron microscopy of a S. lupi female from Hungary showing the eggs in a uterus cross-section. (TIF 2174 kb) [file 13071_2018_3202_MOESM8_ESM.tif]
