# Supplementary material for: Phylogenetic analysis of Spirocerca lupi and Spirocerca vulpis reveal high genetic diversity and intra-individual variation
Source: Parasit Vectors. 2018 Dec 14;11:639. doi: 10.1186/s13071-018-3202-0 (PMC6295112; doi:10.1186/s13071-018-3202-0)
Supplement: Supplementary file 3 — Table S1. Pairwise nucleotide distance of the 18S gene (1611 bp fragment) expressed as percentages between specimens of Spirocerca spp. and other nematodes of the order Spirurida. (DOCX 18 kb) [file 13071_2018_3202_MOESM3_ESM.docx]

**Additional file 3: Table S1.** (DOC 19 kb) Pairwise nucleotide distance of the 18S gene (1611 bp fragment) expressed as percentages between specimens *Spirocerca* spp. and other nematodes of the Spiruridae order.

| No. | Species/host/location | 1 | 2 | 3 | 4 | 5 | 6 | 7 | 8 | 9 | 10 | 11 | 12 | 13 | 14 | 15 |
| --- | --- | --- | --- | --- | --- | --- | --- | --- | --- | --- | --- | --- | --- | --- | --- | --- |
| 1 | *Spirocerca lupi* / Dog / Israel |  |  |  |  |  |  |  |  |  |  |  |  |  |  |  |
| 2 | *Spirocerca lupi* / Dog / Hungary | 0.187 |  |  |  |  |  |  |  |  |  |  |  |  |  |  |
| 3 | *Spirocerca vulpis* / Red fox / Bosnia and Herzegovina | 0.062 | 0.062 |  |  |  |  |  |  |  |  |  |  |  |  |  |
| 4 | Q674750.1 *Spirocerca lupi* / Dog / South Africa | 0.000 | 0.512 | 0.256 |  |  |  |  |  |  |  |  |  |  |  |  |
| 5 | AY751497.1 *Spirocerca lupi* / Dog / USA | 0.312 | 0.375 | 0.313 | 0.254 |  |  |  |  |  |  |  |  |  |  |  |
| 6 | AY751498.1 *Spirocerca* sp. / Long island fox / USA | 1.784 | 1.849 | 1.721 | 0.254 | 1.850 |  |  |  |  |  |  |  |  |  |  |
| 7 | AB495401.2 *Gongylonema pulchrum* / Black-caped squirrel monkey / Nepal | 7.787 | 7.792 | 7.576 | 4.655 | 7.797 | 9.372 |  |  |  |  |  |  |  |  |  |
| 8 | AB646109.1 *Gongylonema nepalensis* / Water buffalo / Nepal | 7.783 | 7.789 | 7.572 | 4.364 | 7.792 | 9.367 | 0.251 |  |  |  |  |  |  |  |  |
| 9 | LC316613.1 *Oxyspirura petrowi* / Northern bobwhite quail / USA | 8.077 | 8.159 | 7.868 | 3.783 | 8.160 | 6.275 | 6.441 | 6.588 |  |  |  |  |  |  |  |
| 10 | AY843436.1 *Wuchereria bancrofti* / NI / NI | 6.576 | 6.656 | 6.371 | 4.630 | 6.657 | 8.034 | 4.283 | 4.278 | 5.151 |  |  |  |  |  |  |
| 11 | XR 002251421.1 *Loa loa* / NI / NI | 6.131 | 6.209 | 5.927 | 4.054 | 6.209 | 7.572 | 4.065 | 4.060 | 4.812 | 0.700 |  |  |  |  |  |
| 12 | AB973229.1 *Dirofilaria repens* / Human / Japan | 6.131 | 6.209 | 5.927 | 4.341 | 6.210 | 7.572 | 4.136 | 4.132 | 4.669 | 0.763 | 0.315 |  |  |  |  |
| 13 | AF182647.1 *Dirofilaria immitis* / Dog / USA | 9.070 | 9.168 | 8.980 | 4.156 | 9.173 | 8.893 | 6.868 | 6.954 | 4.586 | 3.041 | 3.226 | 3.045 |  |  |  |
| 14 | AF227233.1 *Litomosoides sigmodontis* / NI / NI | 7.941 | 8.102 | 7.810 | 5.475 | 8.026 | 9.292 | 5.981 | 6.203 | 7.242 | 4.361 | 3.525 | 3.525 | 5.128 |  |  |
| 15 | AB538282.1 *Thelazia callipaeda* / Human / Japan | 7.412 | 7.499 | 7.191 | 6.459 | 7.500 | 7.022 | 8.119 | 7.865 | 6.795 | 5.850 | 5.644 | 5.404 | 4.995 | 8.399 |  |
| 16 | DQ503458.1 *Thelazia lacrymalis* / Horse / USA | 11.199 | 11.289 | 11.067 | 6.118 | 11.292 | 10.076 | 10.186 | 9.934 | 8.282 | 8.376 | 8.113 | 7.876 | 7.163 | 11.143 | 2.854 |
