# Supplementary material for: Phylogenetic analysis of Spirocerca lupi and Spirocerca vulpis reveal high genetic diversity and intra-individual variation
Source: Parasit Vectors. 2018 Dec 14;11:639. doi: 10.1186/s13071-018-3202-0 (PMC6295112; doi:10.1186/s13071-018-3202-0)
Supplement: Supplementary file 5 — Table S2. Pairwise nucleotide differences (%) between cox1 fragment A (317 to 967 bp) haplotypes obtained from S. lupi and S. vulpis, and reference cox1 sequences of Spirocerca spp. and Cylicospirura spp. available on GenBank. (DOCX 22 kb) [file 13071_2018_3202_MOESM5_ESM.docx]

**Additional file 5: Table S2**. (DOC 23 kb) Pairwise nucleotide differences (%) between *cox1* fragment A (317 to 967 bp) haplotypes obtained from *S. lupi* and *S. vulpis,* and reference *cox1* sequences of *Spirocerca* spp. and *Cylicospirura* spp. available from GenBank.

| No. | Species/host/location/haplotype | 1 | 2 | 3 | 4 | 5 | 6 | 7 | 8 | 9 | 10 | 11 | 12 | 13 | 14 | 15 | 16 | 17 | 18 | 19 | 20 | 21 | 22 | 23 | 24 | 25 |
| --- | --- | --- | --- | --- | --- | --- | --- | --- | --- | --- | --- | --- | --- | --- | --- | --- | --- | --- | --- | --- | --- | --- | --- | --- | --- | --- |
| 1 | *Spirocerca* *lupi* / Dog / Israel / A |  |  |  |  |  |  |  |  |  |  |  |  |  |  |  |  |  |  |  |  |  |  |  |  |  |
| 2 | *Spirocerca* *lupi* / Dog / Israel / B | 0.92 |  |  |  |  |  |  |  |  |  |  |  |  |  |  |  |  |  |  |  |  |  |  |  |  |
| 3 | *Spirocerca* *lupi* / Dog / Israel / C | 0.73 | 0.55 |  |  |  |  |  |  |  |  |  |  |  |  |  |  |  |  |  |  |  |  |  |  |  |
| 4 | *Spirocerca* *lupi* / Dog / Israel / E | 0.73 | 0.55 | 0.73 |  |  |  |  |  |  |  |  |  |  |  |  |  |  |  |  |  |  |  |  |  |  |
| 5 | *Spirocerca* *lupi* / Dog / Israel / H | 1.10 | 0.92 | 1.10 | 0.37 |  |  |  |  |  |  |  |  |  |  |  |  |  |  |  |  |  |  |  |  |  |
| 6 | *Spirocerca* *lupi* / Dog / South Africa / I | 2.75 | 2.56 | 2.75 | 2.38 | 2.75 |  |  |  |  |  |  |  |  |  |  |  |  |  |  |  |  |  |  |  |  |
| 7 | *Spirocerca* *lupi* / Dog / South Africa / J | 2.93 | 2.75 | 2.93 | 2.56 | 2.93 | 0.55 |  |  |  |  |  |  |  |  |  |  |  |  |  |  |  |  |  |  |  |
| 8 | *Spirocerca* *lupi* / Dog / South Africa / K | 2.56 | 2.38 | 2.56 | 2.20 | 2.56 | 0.55 | 0.73 |  |  |  |  |  |  |  |  |  |  |  |  |  |  |  |  |  |  |
| 9 | *Spirocerca* *lupi* / Dog / South Africa / L | 2.38 | 1.83 | 2.01 | 2.01 | 2.38 | 2.93 | 3.11 | 2.75 |  |  |  |  |  |  |  |  |  |  |  |  |  |  |  |  |  |
| 10 | *Spirocerca* *lupi* / Dog / India / M | 3.11 | 2.93 | 3.11 | 2.75 | 3.11 | 2.56 | 2.75 | 2.38 | 3.66 |  |  |  |  |  |  |  |  |  |  |  |  |  |  |  |  |
| 11 | *Spirocerca* *lupi* / Dog / India / N | 2.75 | 2.56 | 2.75 | 2.38 | 2.75 | 2.20 | 2.38 | 2.01 | 3.30 | 0.37 |  |  |  |  |  |  |  |  |  |  |  |  |  |  |  |
| 12 | *Spirocerca* *lupi* / Dog / India / O | 3.11 | 2.93 | 3.11 | 2.75 | 3.11 | 2.56 | 2.75 | 2.38 | 3.66 | 0.37 | 0.37 |  |  |  |  |  |  |  |  |  |  |  |  |  |  |
| 13 | *Spirocerca* *lupi* / Dog / India / P | 2.93 | 2.75 | 2.93 | 2.56 | 2.93 | 2.38 | 2.56 | 2.20 | 3.11 | 0.55 | 0.18 | 0.55 |  |  |  |  |  |  |  |  |  |  |  |  |  |
| 14 | *Spirocerca* *lupi* / Dog / India / Q | 3.30 | 3.11 | 3.30 | 2.93 | 3.30 | 2.75 | 2.93 | 2.56 | 3.48 | 0.55 | 0.55 | 0.18 | 0.37 |  |  |  |  |  |  |  |  |  |  |  |  |
| 15 | NC021135.1 *Spirocerca* *lupi* / Dog / China | 3.11 | 2.93 | 3.11 | 2.75 | 3.11 | 2.56 | 2.75 | 2.38 | 3.66 | 0.37 | 0.37 | 0.37 | 0.55 | 0.55 |  |  |  |  |  |  |  |  |  |  |  |
| 16 | *Spirocerca* *lupi* / Dog / Hungary / AA | 6.24 | 6.06 | 5.50 | 5.87 | 6.24 | 6.42 | 6.61 | 6.24 | 6.79 | 6.42 | 6.42 | 6.42 | 6.24 | 6.24 | 6.42 |  |  |  |  |  |  |  |  |  |  |
| 17 | *Spirocerca* *lupi* / Dog / Hungary / CA | 6.04 | 5.86 | 5.31 | 5.68 | 6.04 | 6.59 | 6.78 | 6.41 | 6.59 | 6.23 | 6.23 | 6.23 | 6.04 | 6.04 | 6.23 | 0.18 |  |  |  |  |  |  |  |  |  |
| 18 | *Spirocerca* *vulpis* / Red fox / Spain / S | 8.79 | 8.61 | 8.42 | 8.79 | 8.79 | 8.06 | 8.24 | 8.24 | 8.61 | 9.34 | 9.16 | 9.34 | 8.97 | 9.16 | 8.97 | 8.99 | 9.16 |  |  |  |  |  |  |  |  |
| 19 | *Spirocerca* *vulpis* / Red fox / Spain / T | 8.61 | 8.79 | 8.24 | 8.97 | 8.97 | 8.24 | 8.42 | 8.42 | 8.79 | 9.34 | 9.16 | 9.34 | 8.97 | 9.16 | 8.97 | 8.62 | 8.79 | 1.28 |  |  |  |  |  |  |  |
| 20 | *Spirocerca* *vulpis* / Red fox / Spain / U | 7.88 | 8.06 | 7.51 | 8.24 | 8.24 | 7.51 | 7.69 | 7.69 | 8.42 | 8.79 | 8.61 | 8.79 | 8.42 | 8.61 | 8.42 | 8.26 | 8.42 | 1.65 | 1.10 |  |  |  |  |  |  |
| 21 | *Spirocerca* *vulpis* / Red fox / Italy/ X | 8.24 | 8.79 | 8.24 | 8.61 | 8.61 | 7.88 | 8.06 | 8.06 | 8.79 | 8.97 | 8.79 | 8.97 | 8.61 | 8.79 | 8.61 | 8.62 | 8.79 | 1.65 | 0.73 | 1.10 |  |  |  |  |  |
| 22 | *Spirocerca* *lupi* / Dog / Bosnia and Herzegovina / EA | 8.24 | 8.79 | 8.24 | 8.61 | 8.61 | 7.88 | 8.06 | 8.06 | 8.79 | 8.97 | 8.79 | 8.97 | 8.61 | 8.79 | 8.61 | 8.62 | 8.79 | 1.28 | 0.37 | 1.10 | 0.37 |  |  |  |  |
| 23 | KJ605487.1 *Spirocerca* sp. / Red fox / Denmark | 8.24 | 8.79 | 8.24 | 8.61 | 8.61 | 7.88 | 8.06 | 8.06 | 8.79 | 8.97 | 8.79 | 8.97 | 8.61 | 8.79 | 8.61 | 8.62 | 8.79 | 1.28 | 0.37 | 1.10 | 0.37 | 0.00 |  |  |  |
| 24 | KF719952.1 *Cylicospirura petrowi* / Germany | 10.99 | 10.81 | 10.99 | 10.26 | 10.26 | 10.44 | 10.62 | 9.89 | 11.54 | 11.17 | 10.81 | 11.17 | 10.99 | 11.36 | 10.81 | 10.09 | 10.26 | 12.09 | 12.27 | 11.17 | 11.90 | 11.90 | 11.90 |  |  |
| 25 | GQ342967.1 *Cylicospirura felineus* / USA | 10.62 | 10.07 | 10.26 | 10.26 | 10.62 | 10.26 | 10.44 | 10.44 | 10.44 | 10.62 | 10.26 | 10.62 | 10.44 | 10.81 | 10.62 | 11.19 | 10.99 | 11.54 | 11.36 | 10.62 | 11.36 | 11.36 | 11.36 | 7.69 |  |
| 26 | GQ342968.1 *Cylicospirura subaequalis* / USA | 10.81 | 10.62 | 10.81 | 10.44 | 10.26 | 10.44 | 10.62 | 10.62 | 10.81 | 11.36 | 10.99 | 11.36 | 10.81 | 11.17 | 11.36 | 10.46 | 10.26 | 10.44 | 10.26 | 9.52 | 10.26 | 10.26 | 10.26 | 10.62 | 10.44 |
