# Supplementary material for: Phylogenetic analysis of Spirocerca lupi and Spirocerca vulpis reveal high genetic diversity and intra-individual variation
Source: Parasit Vectors. 2018 Dec 14;11:639. doi: 10.1186/s13071-018-3202-0 (PMC6295112; doi:10.1186/s13071-018-3202-0)
Supplement: Supplementary file 6 — Table S3.Pairwise nucleotide differences (%) between cox1 fragment B (807 to 1201 bp) haplotypes obtained from S. lupi and S. vulpis, and cox1 reference sequences of S. lupi and D. immitis available on GenBank. (DOCX 31 kb) [file 13071_2018_3202_MOESM6_ESM.docx]

**Additional file 6: Table S3.** (DOC 32 kb) Pairwise nucleotide differences (%) between *cox1* fragment B (807 to 1201 bp) haplotypes obtained from *S. lupi* and *S. vulpis*, and *cox1* reference sequences of *S. lupi* and *D. immitis* available from GenBank.

| No. | Species/host/location/haplotype | 1 | 2 | 3 | 4 | 5 | 6 | 7 | 8 | 9 | 10 | 11 | 12 | 13 | 14 | 15 | 16 | 17 | 18 | 19 | 20 | 21 | 22 | 23 | 24 | 25 | 26 | 27 | 28 | 29 | 30 | 31 | 32 | 33 |
| --- | --- | --- | --- | --- | --- | --- | --- | --- | --- | --- | --- | --- | --- | --- | --- | --- | --- | --- | --- | --- | --- | --- | --- | --- | --- | --- | --- | --- | --- | --- | --- | --- | --- | --- |
| 1 | *Spirocerca lupi* / Dog / Israel / A complete |  |  |  |  |  |  |  |  |  |  |  |  |  |  |  |  |  |  |  |  |  |  |  |  |  |  |  |  |  |  |  |  |  |
| 2 | *Spirocerca lupi* / Dog / Israel D complete | 1.16 |  |  |  |  |  |  |  |  |  |  |  |  |  |  |  |  |  |  |  |  |  |  |  |  |  |  |  |  |  |  |  |  |
| 3 | *Spirocerca lupi* / Dog / Israel / G complete | 0.87 | 2.02 |  |  |  |  |  |  |  |  |  |  |  |  |  |  |  |  |  |  |  |  |  |  |  |  |  |  |  |  |  |  |  |
| 4 | *Spirocerca lupi* / Dog / India / M complete | 3.76 | 4.34 | 3.47 |  |  |  |  |  |  |  |  |  |  |  |  |  |  |  |  |  |  |  |  |  |  |  |  |  |  |  |  |  |  |
| 5 | NC021135.1 *Spirocerca lupi* / Dog / China | 4.05 | 4.62 | 3.76 | 0.29 |  |  |  |  |  |  |  |  |  |  |  |  |  |  |  |  |  |  |  |  |  |  |  |  |  |  |  |  |  |
| 6 | *Spirocerca lupi* / Dog / South Africa / I complete | 3.47 | 4.34 | 3.76 | 4.34 | 4.62 |  |  |  |  |  |  |  |  |  |  |  |  |  |  |  |  |  |  |  |  |  |  |  |  |  |  |  |  |
| 7 | *Spirocerca lupi* / Dog / South Africa / J complete | 3.76 | 4.62 | 4.05 | 4.05 | 4.34 | 0.29 |  |  |  |  |  |  |  |  |  |  |  |  |  |  |  |  |  |  |  |  |  |  |  |  |  |  |  |
| 8 | *Spirocerca lupi* / Dog / South Africa / K complete | 3.76 | 4.62 | 4.05 | 4.05 | 4.34 | 0.87 | 0.58 |  |  |  |  |  |  |  |  |  |  |  |  |  |  |  |  |  |  |  |  |  |  |  |  |  |  |
| 9 | *Spirocerca lupi* / Dog / South Africa / L complete | 0.58 | 1.73 | 1.45 | 3.18 | 3.47 | 2.89 | 3.18 | 3.18 |  |  |  |  |  |  |  |  |  |  |  |  |  |  |  |  |  |  |  |  |  |  |  |  |  |
| 10 | KY634870.1 *Spirocerca lupi* / Andean fox / Peru / F2 | 0.87 | 2.02 | 1.73 | 4.05 | 4.34 | 4.34 | 4.62 | 4.62 | 1.45 |  |  |  |  |  |  |  |  |  |  |  |  |  |  |  |  |  |  |  |  |  |  |  |  |
| 11 | KY634869.1 *Spirocerca lupi* isolate / Andean fox / Peru / M1 | 0.58 | 1.73 | 1.45 | 3.76 | 4.05 | 4.05 | 4.34 | 4.34 | 1.16 | 0.29 |  |  |  |  |  |  |  |  |  |  |  |  |  |  |  |  |  |  |  |  |  |  |  |
| 12 | KY634868.1 *Spirocerca lupi* isolate / Andean fox / Peru / F1 | 0.87 | 2.02 | 1.73 | 4.05 | 4.34 | 4.34 | 4.62 | 4.62 | 1.45 | 0.00 | 0.29 |  |  |  |  |  |  |  |  |  |  |  |  |  |  |  |  |  |  |  |  |  |  |
| 13 | KY495505.1 *Spirocerca lupi* / Dog / South Africa / haplotype 24 | 4.34 | 5.20 | 4.62 | 4.62 | 4.91 | 0.87 | 0.58 | 1.16 | 3.76 | 5.20 | 4.91 | 5.20 |  |  |  |  |  |  |  |  |  |  |  |  |  |  |  |  |  |  |  |  |  |
| 14 | KY495504.1 *Spirocerca lupi* / Dog / South Africa / haplotype 23 | 4.34 | 5.20 | 4.62 | 4.62 | 4.91 | 0.87 | 0.58 | 1.16 | 3.76 | 5.20 | 4.91 | 5.20 | 1.16 |  |  |  |  |  |  |  |  |  |  |  |  |  |  |  |  |  |  |  |  |
| 15 | KY495503.1 *Spirocerca lupi* / Dog / South Africa / haplotype 22 | 4.05 | 4.91 | 4.34 | 4.34 | 4.62 | 0.58 | 0.29 | 0.87 | 3.47 | 4.91 | 4.62 | 4.91 | 0.87 | 0.87 |  |  |  |  |  |  |  |  |  |  |  |  |  |  |  |  |  |  |  |
| 16 | KY495502.1 *Spirocerca lupi* / Dog / South Africa / haplotype 21 | 4.05 | 4.91 | 4.34 | 4.34 | 4.62 | 0.58 | 0.29 | 0.87 | 3.47 | 4.91 | 4.62 | 4.91 | 0.87 | 0.87 | 0.58 |  |  |  |  |  |  |  |  |  |  |  |  |  |  |  |  |  |  |
| 17 | KY495501.1 *Spirocerca lupi* / Dog / South Africa / haplotype 20 | 4.05 | 4.91 | 4.34 | 4.34 | 4.62 | 0.58 | 0.29 | 0.87 | 3.47 | 4.91 | 4.62 | 4.91 | 0.87 | 0.87 | 0.58 | 0.58 |  |  |  |  |  |  |  |  |  |  |  |  |  |  |  |  |  |
| 18 | KY495500.1 *Spirocerca lupi* / Dog / South Africa / haplotype 19 | 4.05 | 4.91 | 4.34 | 4.34 | 4.62 | 0.58 | 0.29 | 0.87 | 3.47 | 4.91 | 4.62 | 4.91 | 0.87 | 0.87 | 0.58 | 0.58 | 0.58 |  |  |  |  |  |  |  |  |  |  |  |  |  |  |  |  |
| 19 | KY495499.1 *Spirocerca lupi* / Dog / South Africa / haplotype 18 | 4.05 | 4.91 | 4.34 | 4.34 | 4.62 | 0.58 | 0.29 | 0.87 | 3.47 | 4.91 | 4.62 | 4.91 | 0.87 | 0.87 | 0.58 | 0.58 | 0.58 | 0.58 |  |  |  |  |  |  |  |  |  |  |  |  |  |  |  |
| 20 | KY495498.1 *Spirocerca lupi* / Dog / South Africa / haplotype 17 | 4.34 | 5.20 | 4.62 | 4.62 | 4.91 | 0.87 | 0.58 | 1.16 | 3.76 | 5.20 | 4.91 | 5.20 | 1.16 | 1.16 | 0.87 | 0.87 | 0.87 | 0.87 | 0.87 |  |  |  |  |  |  |  |  |  |  |  |  |  |  |
| 21 | KY495497.1 *Spirocerca lupi* / Dog / South Africa / haplotype 16 | 4.05 | 4.91 | 4.34 | 4.34 | 4.62 | 0.58 | 0.29 | 0.87 | 3.47 | 4.91 | 4.62 | 4.91 | 0.87 | 0.87 | 0.58 | 0.58 | 0.58 | 0.58 | 0.29 | 0.87 |  |  |  |  |  |  |  |  |  |  |  |  |  |
| 22 | KY495496.1 *Spirocerca lupi* / Dog / South Africa / haplotype 15 | 4.05 | 4.91 | 4.34 | 4.34 | 4.62 | 1.16 | 0.87 | 0.87 | 3.47 | 4.91 | 4.62 | 4.91 | 1.45 | 1.45 | 1.16 | 1.16 | 1.16 | 1.16 | 1.16 | 1.45 | 1.16 |  |  |  |  |  |  |  |  |  |  |  |  |
| 23 | KY495495.1 *Spirocerca lupi* / Black backed jackal / South Africa/ haplotype 14 | 3.18 | 4.05 | 3.47 | 3.47 | 3.76 | 0.87 | 0.58 | 0.58 | 2.60 | 4.05 | 3.76 | 4.05 | 1.16 | 1.16 | 0.87 | 0.87 | 0.87 | 0.87 | 0.87 | 1.16 | 0.87 | 0.87 |  |  |  |  |  |  |  |  |  |  |  |
| 24 | KY495494.1 *Spirocerca lupi* / Black backed jackal / South Africa/ haplotype 13 | 3.76 | 4.62 | 4.05 | 4.05 | 4.34 | 0.87 | 0.58 | 0.58 | 3.18 | 4.62 | 4.34 | 4.62 | 1.16 | 1.16 | 0.87 | 0.87 | 0.87 | 0.87 | 0.87 | 1.16 | 0.87 | 0.87 | 0.58 |  |  |  |  |  |  |  |  |  |  |
| 25 | KY495493.1 *Spirocerca lupi* / Black backed jackal / South Africa/ haplotype 12 | 3.76 | 4.62 | 4.05 | 4.05 | 4.34 | 0.29 | 0.00 | 0.58 | 3.18 | 4.62 | 4.34 | 4.62 | 0.58 | 0.58 | 0.29 | 0.29 | 0.29 | 0.29 | 0.29 | 0.58 | 0.29 | 0.87 | 0.58 | 0.58 |  |  |  |  |  |  |  |  |  |
| 26 | *Spirocerca lupi* / Dog / Hungary / AA complete | 6.36 | 6.36 | 6.65 | 7.80 | 8.09 | 8.09 | 7.80 | 7.80 | 6.36 | 6.65 | 6.94 | 6.65 | 8.38 | 8.38 | 8.09 | 8.09 | 8.09 | 8.09 | 8.09 | 7.80 | 8.09 | 8.09 | 7.51 | 7.80 | 7.80 |  |  |  |  |  |  |  |  |
| 27 | *Spirocerca lupi* / Dog / Hungary / BA complete | 6.65 | 6.07 | 6.94 | 6.94 | 7.23 | 8.09 | 7.80 | 7.80 | 6.65 | 6.94 | 7.23 | 6.94 | 8.38 | 8.38 | 8.09 | 8.09 | 8.09 | 8.09 | 8.09 | 7.80 | 8.09 | 8.09 | 7.23 | 7.80 | 7.80 | 0.87 |  |  |  |  |  |  |  |
| 28 | *Spirocerca lupi* / Dog / Hungary / CA complete | 6.65 | 6.07 | 6.94 | 7.51 | 7.80 | 8.38 | 8.09 | 8.09 | 6.65 | 6.94 | 7.23 | 6.94 | 8.67 | 8.67 | 8.38 | 8.38 | 8.38 | 8.38 | 8.38 | 8.09 | 8.38 | 8.38 | 7.80 | 8.09 | 8.09 | 0.58 | 0.58 |  |  |  |  |  |  |
| 29 | *Spirocerca vulpis* / Red fox / Spain / S complete | 6.36 | 6.36 | 6.65 | 7.23 | 7.51 | 7.80 | 8.09 | 8.67 | 6.36 | 6.07 | 6.36 | 6.07 | 8.67 | 8.67 | 8.38 | 8.38 | 8.38 | 8.38 | 8.38 | 8.67 | 8.38 | 8.96 | 8.09 | 8.67 | 8.09 | 8.09 | 7.23 | 7.80 |  |  |  |  |  |
| 30 | *Spirocerca vulpis* / Red fox / Spain / T complete | 6.36 | 6.36 | 6.65 | 7.80 | 8.09 | 7.80 | 8.09 | 8.67 | 6.36 | 6.07 | 6.36 | 6.07 | 8.67 | 8.67 | 8.38 | 8.38 | 8.38 | 8.38 | 8.38 | 8.09 | 8.38 | 8.96 | 8.09 | 8.67 | 8.09 | 8.09 | 7.23 | 7.80 | 1.16 |  |  |  |  |
| 31 | *Spirocerca vulpis* / Red fox / Spain / U complete | 6.36 | 6.36 | 6.65 | 7.80 | 8.09 | 7.80 | 8.09 | 8.67 | 6.36 | 6.07 | 6.36 | 6.07 | 8.67 | 8.67 | 8.38 | 8.38 | 8.38 | 8.38 | 8.38 | 8.09 | 8.38 | 8.96 | 8.09 | 8.67 | 8.09 | 8.09 | 7.23 | 7.80 | 1.16 | 0.00 |  |  |  |
| 32 | *Spirocerca vulpis* / Red fox / Italy / X complete | 6.36 | 6.36 | 6.65 | 7.80 | 8.09 | 7.80 | 8.09 | 8.67 | 6.36 | 6.07 | 6.36 | 6.07 | 8.67 | 8.67 | 8.38 | 8.38 | 8.38 | 8.38 | 8.38 | 8.09 | 8.38 | 8.96 | 8.09 | 8.67 | 8.09 | 8.09 | 7.23 | 7.80 | 1.16 | 0.00 | 0.00 |  |  |
| 33 | *Spirocerca vulpis* / Red fox / Bosnia / EA complete | 6.65 | 6.65 | 6.94 | 8.09 | 8.38 | 8.09 | 8.38 | 8.96 | 6.65 | 6.36 | 6.65 | 6.36 | 8.96 | 8.96 | 8.67 | 8.67 | 8.67 | 8.67 | 8.67 | 8.38 | 8.67 | 9.25 | 8.38 | 8.96 | 8.38 | 8.38 | 7.51 | 8.09 | 1.45 | 0.29 | 0.29 | 0.29 |  |
| 34 | NC005305.1 *Dirofilaria immitis* / Dog / Australia | 14.74 | 14.16 | 14.74 | 13.29 | 13.29 | 14.74 | 14.45 | 15.03 | 14.74 | 15.03 | 14.74 | 15.03 | 15.03 | 15.03 | 14.74 | 14.74 | 14.74 | 14.74 | 14.74 | 14.45 | 14.74 | 15.32 | 14.74 | 15.03 | 14.45 | 14.74 | 14.45 | 14.16 | 15.03 | 14.45 | 14.45 | 14.45 | 14.74 |
