# Supplementary material for: Phylogenetic analysis of Spirocerca lupi and Spirocerca vulpis reveal high genetic diversity and intra-individual variation
Source: Parasit Vectors. 2018 Dec 14;11:639. doi: 10.1186/s13071-018-3202-0 (PMC6295112; doi:10.1186/s13071-018-3202-0)
Supplement: Supplementary file 7 — Table S4.Pairwise nucleotide distance (%) in the cox1 (317 to 1201 bp) haplotypes of Spirocerca spp. (DOCX 16 kb) [file 13071_2018_3202_MOESM7_ESM.docx]

**Additional file 7: Table S4.** Pairwise nucleotide distance (%) in the *cox*1 (317 to 1201 bp) haplotypes of *Spirocerca* spp.

| No. | Species/host/location/haplotype | 1 | 2 | 3 | 4 | 5 | 6 | 7 | 8 | 9 | 10 | 11 | 12 | 13 | 14 | 15 | 16 | 17 | 18 | 19 | 20 | 21 | 22 |
| --- | --- | --- | --- | --- | --- | --- | --- | --- | --- | --- | --- | --- | --- | --- | --- | --- | --- | --- | --- | --- | --- | --- | --- |
| 1 | *Spirocerca lupi*/Dog/Israel/A |  |  |  |  |  |  |  |  |  |  |  |  |  |  |  |  |  |  |  |  |  |  |
| 2 | *Spirocerca lupi*/Dog/Israel/B | 0.591 |  |  |  |  |  |  |  |  |  |  |  |  |  |  |  |  |  |  |  |  |  |
| 3 | *Spirocerca lupi*/Dog/Israel/C | 0.473 | 0.355 |  |  |  |  |  |  |  |  |  |  |  |  |  |  |  |  |  |  |  |  |
| 4 | *Spirocerca lupi*/Dog/Israel/E | 0.473 | 0.355 | 0.473 |  |  |  |  |  |  |  |  |  |  |  |  |  |  |  |  |  |  |  |
| 5 | *Spirocerca lupi*/Dog/Israel/G | 0.827 | 0.709 | 0.827 | 0.355 |  |  |  |  |  |  |  |  |  |  |  |  |  |  |  |  |  |  |
| 6 | *Spirocerca lupi*/Dog/South Africa/I | 2.955 | 2.837 | 2.955 | 2.719 | 2.837 |  |  |  |  |  |  |  |  |  |  |  |  |  |  |  |  |  |
| 7 | *Spirocerca lupi*/Dog/South Africa/J | 3.191 | 3.073 | 3.191 | 2.955 | 3.073 | 0.473 |  |  |  |  |  |  |  |  |  |  |  |  |  |  |  |  |
| 8 | *Spirocerca lupi*/Dog/South Africa/K | 3.073 | 2.955 | 3.073 | 2.837 | 2.955 | 0.591 | 0.591 |  |  |  |  |  |  |  |  |  |  |  |  |  |  |  |
| 9 | *Spirocerca lupi*/Dog/South Africa/L | 1.773 | 1.418 | 1.537 | 1.537 | 1.891 | 2.837 | 3.073 | 2.955 |  |  |  |  |  |  |  |  |  |  |  |  |  |  |
| 10 | *Spirocerca lupi*/Dog/India/M | 3.428 | 3.310 | 3.428 | 3.191 | 3.073 | 3.073 | 3.073 | 2.955 | 3.546 |  |  |  |  |  |  |  |  |  |  |  |  |  |
| 11 | *Spirocerca lupi*/Dog/India/N | 3.191 | 3.073 | 3.191 | 2.955 | 2.837 | 2.837 | 2.837 | 2.719 | 3.310 | 0.236 |  |  |  |  |  |  |  |  |  |  |  |  |
| 12 | *Spirocerca lupi*/Dog/India/O | 3.428 | 3.310 | 3.428 | 3.191 | 3.073 | 3.073 | 3.073 | 2.955 | 3.546 | 0.236 | 0.236 |  |  |  |  |  |  |  |  |  |  |  |
| 13 | *Spirocerca lupi*/Dog/India/P | 3.310 | 3.191 | 3.310 | 3.073 | 2.955 | 2.955 | 2.955 | 2.837 | 3.191 | 0.355 | 0.118 | 0.355 |  |  |  |  |  |  |  |  |  |  |
| 14 | *Spirocerca lupi*/Dog/India/Q | 3.546 | 3.428 | 3.546 | 3.310 | 3.191 | 3.191 | 3.191 | 3.073 | 3.428 | 0.355 | 0.355 | 0.118 | 0.236 |  |  |  |  |  |  |  |  |  |
| 15 | NC021135.1 *Spirocerca lupi*/Dog/China | 3.546 | 3.428 | 3.546 | 3.310 | 3.191 | 3.191 | 3.191 | 3.073 | 3.664 | 0.355 | 0.355 | 0.355 | 0.473 | 0.473 |  |  |  |  |  |  |  |  |
| 16 | *Spirocerca lupi*/Dog/Hungary/AA | 6.383 | 6.265 | 5.910 | 6.147 | 6.265 | 6.738 | 6.738 | 6.619 | 6.501 | 6.738 | 6.738 | 6.738 | 6.619 | 6.619 | 6.856 |  |  |  |  |  |  |  |
| 17 | *Spirocerca lupi*/Dog/Hungary/CA | 6.383 | 6.265 | 5.910 | 6.147 | 6.265 | 6.974 | 6.974 | 6.856 | 6.501 | 6.501 | 6.501 | 6.501 | 6.383 | 6.383 | 6.619 | 0.355 |  |  |  |  |  |  |
| 18 | *Spirocerca vulpis*/Red fox/Spain/S | 7.565 | 7.447 | 7.329 | 7.565 | 7.683 | 7.683 | 7.920 | 8.038 | 7.447 | 8.156 | 8.038 | 8.156 | 7.920 | 8.038 | 8.038 | 8.629 | 8.629 |  |  |  |  |  |
| 19 | *Spirocerca vulpis*/Red fox/Spain/T | 7.329 | 7.447 | 7.092 | 7.565 | 7.683 | 7.683 | 7.920 | 8.038 | 7.447 | 8.274 | 8.156 | 8.274 | 8.038 | 8.156 | 8.156 | 8.511 | 8.511 | 1.182 |  |  |  |  |
| 20 | *Spirocerca vulpis*/Red fox/Spain/U | 6.856 | 6.974 | 6.619 | 7.092 | 7.210 | 7.210 | 7.447 | 7.565 | 7.210 | 7.920 | 7.801 | 7.920 | 7.683 | 7.801 | 7.801 | 8.274 | 8.274 | 1.418 | 0.709 |  |  |  |
| 21 | *Spirocerca vulpis*/Red fox/Italy/X | 7.092 | 7.447 | 7.092 | 7.329 | 7.447 | 7.447 | 7.683 | 7.801 | 7.447 | 8.038 | 7.920 | 8.038 | 7.801 | 7.920 | 7.920 | 8.511 | 8.511 | 1.418 | 0.473 | 0.709 |  |  |
| 22 | *Spirocerca vulpis*/Red fox/Bosnia and Herzegovina/FA | 7.210 | 7.565 | 7.210 | 7.447 | 7.565 | 7.565 | 7.801 | 7.920 | 7.565 | 8.156 | 8.038 | 8.156 | 7.920 | 8.038 | 8.038 | 8.629 | 8.629 | 1.300 | 0.355 | 0.827 | 0.355 |  |
| 23 | AJ537512.1:2268-3914 *Dirofilaria immitis*/Dog/Australia | 14.539 | 14.184 | 14.066 | 14.066 | 14.066 | 13.357 | 13.475 | 13.475 | 14.303 | 13.712 | 13.475 | 13.712 | 13.593 | 13.830 | 13.712 | 13.593 | 13.475 | 14.184 | 13.948 | 13.475 | 13.948 | 14.066 |
